# *Schistosoma mansoni* excretory-secretory products induce protein kinase signalling, hyperkinesia, and stem cell proliferation in the opposite sex

**DOI:** 10.1038/s42003-023-05333-9

**Published:** 2023-09-26

**Authors:** Eman M. N. Shakir, Gabriel Rinaldi, Ruth S. Kirk, Anthony J. Walker

**Affiliations:** 1 Molecular Parasitology Laboratory, School of Life Sciences Pharmacy and Chemistry, Kingston University, Penrhyn Road, Kingston upon Thames, KT1 2EE UK; 2 Wellcome Sanger Institute, Wellcome Genome Campus, Hinxton, Cambridge, CB10 1SA UK; 3 Department of Life Sciences, Edward Llwyd Building, Aberystwyth University, Aberystwyth, SY23 3DA UK

**Keywords:** Parasite physiology, Parasite biology, Cell signalling

## Abstract

Adult male and female schistosomes *in copula* dwell within human blood vessels and lay eggs that cause the major Neglected Tropical Disease human schistosomiasis. How males and females communicate to each other is poorly understood; however, male-female physical interaction is known to be important. Here, we investigate whether excretory-secretory products (ESPs), released into the external milieu by mature *Schistosoma mansoni*, might induce responses in the opposite sex. We demonstrate that ESPs adhere to the surface of opposite sex worms inducing the activation of extracellular signal-regulated kinase (ERK) and p38 mitogen-activated protein kinase (p38 MAPK) pathways, particularly in the parasite tegument. Furthermore, we show that mature worms stimulated signalling in juvenile worms. Strikingly, we demonstrate that ESPs from the opposite sex promote stem cell proliferation, in an ERK- and p38 MAPK-dependent manner, in the tegument and within the testes of males, and the ovaries and vitellaria of females. Hyperkinesia also occurs following opposite sex ESP exposure. Our findings support the hypothesis that male and female schistosomes may communicate over distance to modulate key processes underlying worm development and disease progression, opening unique avenues for schistosomiasis control.

## Abstract (web summary)

Mature *Schistosoma mansoni* worms release excretory secretory products (ESPs) which modulate key processes in worms of the opposite sex via the ERK and p38 MAPK pathways, indicating that male and female schistosomes can communicate over distances.

## Introduction

Schistosomes are flatworm parasites that infect >240 million people globally, ~90% of whom are in Africa, responsible for substantial morbidity and mortality^1,2^. After emerging from intermediate host snails, the free-living larval cercariae penetrate human skin, become schistosomules, burrow into the vasculature, and navigate within the circulatory system feeding on red blood cells. Once the parasites reach the branches of the hepatic portal vein, the males and females pair-up and complete the maturation into adult worms^3,4^, which produce and release hundreds of eggs per day^5^. With sustained pairing, oviposition continues throughout the worms’ entire life which can span several decades^6,7^. The eggs are destined to be voided in the faeces or urine (depending on the species) after extravasation, enabling parasite transmission *via* the snail host^3^. However, only ~50% of the laid eggs are believed to exit the host and the remaining get swept back with the blood flow to the liver or spleen where they become lodged, eliciting immune responses that ultimately drive granuloma formation and fibrosis, the hallmarks of human schistosomiasis^8,9^.

The sexual biology of schistosomes is unique among flatworm trematodes given their genetically determined dioecious nature, with homogametic males (2n = 16, ZZ) and heterogametic females (2n = 16, ZW)^10^. The muscular male worm holds the slenderer female in its gynaecophoral canal to facilitate the transfer of sperm. Whereas the mature male schistosome possesses a simple reproductive system comprising several testicular lobes and a vesicle for temporary sperm storage, the female worm has a complex reproductive system that includes the ovary, a seminal receptacle, the oviduct, Mehlis’s gland, an ootype, uterus, and extensive vitellaria^11^. The complex vitellaria comprises progenitor S1 cells that differentiate to S4 cells that possess vitelline and lipid droplets that form the eggshell; these S4 cells merge with a fertilised oocyte in the vitello-oviduct and are transferred to the ootype where egg biogenesis begins^11^. Germline stem cells and vitellocytes are thus crucial for the development and maintenance of reproductive tissues and egg production^12–14^. In addition, somatic stem cells are vital for the maintenance of the worm tegument^15^, which plays a key role in processes such as glucose uptake, immune evasion, and excretion^16–18^.

It has been well established that schistosome reproductive maturation relies strongly on the physical interaction between male and female worms; in particular, the pairing with a male schistosome is critical for reproductive maturation of the female worm^19,20^. Female worms from unisexual infections display underdeveloped Mehlis’s glands, ovaries and vitellaria^21^. The discovery almost four decades ago that segments of male worms can stimulate localised female reproductive development, further highlights the physical contact between sexes as a vital cue^22,23^. A recently reported male-derived di-peptide, produced following physical contact with the female, has been found to regulate the sexual maturation of the latter; notably, immature females also matured independently of males when exposed to the male-derived dipeptide^24^. However, the possibility that molecules released by male and female schistosomes into the external milieu might regulate schistosome development and function through remote signalling (i.e., not pairing dependent) has not been explored and cannot be ruled out a priori.

Using a range of biochemical techniques and confocal microscopy, we demonstrate that excretory-secretory products (ESPs) from male and female *S. mansoni* bind to the tegument of opposite sex worms and stimulate the activation of extracellular signal regulated kinase (ERK) and p38 mitogen-activated kinase (p38 MAPK) pathways, within the tegument and reproductive organs. We further reveal that ESPs from the opposite sex induce schistosome hyperkinesia and promote the proliferation of stem cells, within the testes of males, the ovaries and vitellaria of females, and the tegument of either sex, in an ERK- and p38 MAPK-dependent manner. Collectively, and to the best of our knowledge, this study reveals a novel mechanism of schistosome intersex communication involved in worm development and behaviour regulation.

## Results

### Parasite-derived excretory-secretory products (ESPs) modulate cell signalling in opposite sex worms

**Fig. 1:**
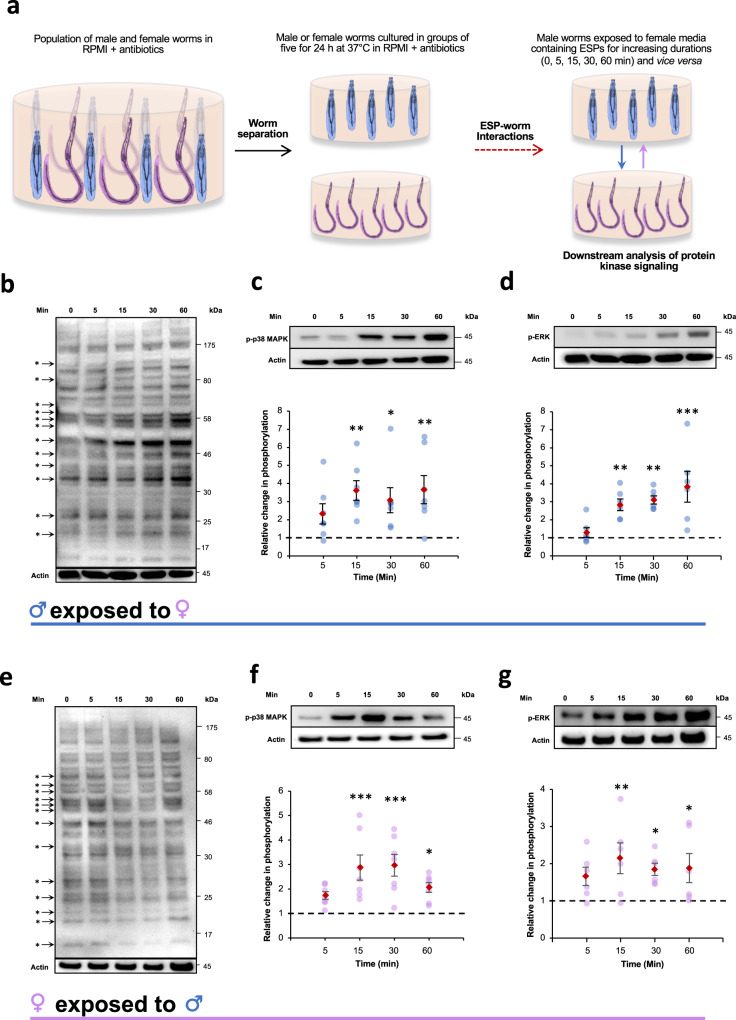
Adult *S. mansoni* ESPs induce protein kinase signalling in opposite sex worms. **a** Schematic depicting the adult worm exposure protocol. **b**–**d** Adult male worms, or **e**–**g** adult female worms, were exposed to 24 h culture media containing ESPs from opposite sex adult worms and processed at the indicated time points. The worm proteins were extracted, and equal protein amounts processed for western blotting with anti-phospho- S/T/Y, -p38 MAPK, or -ERK antibodies, respectively. Apparent changes in S/T/Y phosphorylation are indicated (*). Blots were re-probed for actin as loading control. Mean relative change in phosphorylation (±S.E.M; n ≥ 5 biological replicates) in worms was calculated (graphs) over time, against control 0 min values (assigned a value of 1, dotted line) based on band intensity analysis after normalisation against actin; *p ≤ 0.05, **p ≤ 0.01, and ***p ≤ 0.001 (ANOVA), when compared to control values.

To evaluate the effect of ESPs released by adult male or female parasites on signalling in adults of the opposite sex, worms obtained at day 45 post-infection (D45) were separated into groups of five males or five females and cultured overnight in RPMI without serum. The following day, the media were exchanged between the cultures to perform opposite sex ESP exposures. Thereafter, the worms were collected at different time points and processed for western blotting (Fig. 1a). Initial probing with anti-phospho serine/threonine/tyrosine (pS/pT/pY) antibodies revealed that global protein phosphorylation in male worms exposed to female ESPs changed over time; in particular, phosphorylation of ~12 proteins of distinct molecular weights appeared to increase or decrease within 60 min, with some changes observed as early as 5 min after ESP exposure (Fig. 1b). These results suggest that several protein kinase pathways may be temporally modulated by exposure to opposite sex ESPs. Encouraged by these findings, we performed additional exposures of male adult worms followed by western blotting with a panel of four antibodies (anti-phospho p44/p42 MAPK (ERK), anti-phospho p38 MAPK, anti-phospho PKA-C, and anti-phospho-PKC), which we have previously validated for use with *S. mansoni*^25–31^. Importantly, these antibodies react exclusively with the phosphorylated (activated) form of each target kinase enabling the study of specific pathway activation following treatment. Across multiple experiments (n ≥ 5), both p38 MAPK and ERK were activated in male worms following female ESP exposure, with activation occurring as early as 15 min (p ≤ 0.01) and sustained over 60 min; maximal phosphorylation was ~3.6-fold (p ≤ 0.01) and ~3.9-fold (p ≤ 0.001) at 15 min and 60 min, respectively, when compared to controls (Fig. 1c, d). However, no statistically significant change in PKA or PKC activation in adult males exposed to female ESPs was observed (Supplementary Fig. 1a, b). Intriguingly, when adult female worms were exposed to male ESPs, several (~13) female worm proteins also displayed an apparent increase or decrease in pS, pT, or pY levels over 60 min (Fig. 1e). Furthermore, and similarly to male worms exposed to female ESPs, p38 MAPK and ERK were activated in female worms exposed to male ESPs, with increased phosphorylation occurring after 15 min exposure, and maximal at 30 min (~3-fold; p ≤ 0.001) and 15 min (2.1-fold; p ≤ 0.01), respectively (Fig. 1f, g); PKA and PKC activation remained unaffected (Supplementary Fig. 1c, d). In all cases, and for both male and female worms, the activation status of the protein kinases did not change significantly over 60 min when worms were not exposed to opposite sex ESPs (Supplementary Fig. 2). Given that signalling responses were robustly observed in both sexes for both p38 MAPK and ERK following 15 min ESP exposure (Fig. 1), this time-point was selected for all subsequent experiments investigating protein kinase activation, with time-matched (15 min) unexposed controls included.

We next exposed groups of five adult males to ESPs from a different group of five adult males, carried out a similar experiment with adult females, and determined effects after 15 min exposure. No changes in p38 MAPK or ERK phosphorylation were observed under these conditions (Supplementary Fig. 3). This was anticipated as male or female worms were cultured in unisexual groups of five, and any signalling between same sex worms should be reflected in basal phosphorylation levels within the group. Importantly, however, these findings support that the observed ESP-mediated activation of p38 MAPK and ERK (Fig. 1) is an inter-, rather than intra-sex response.

**Fig. 2:**
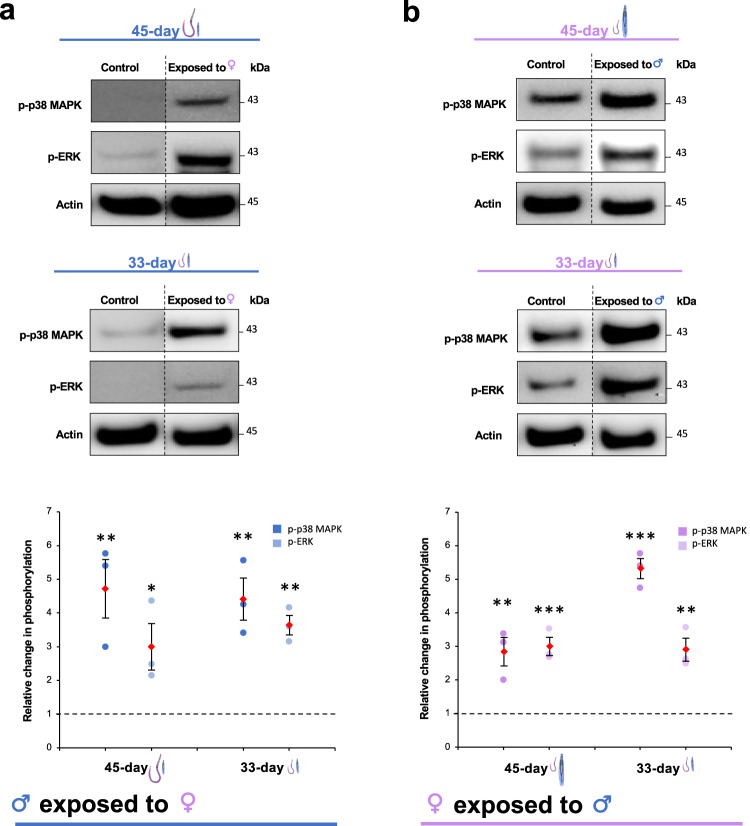
ESPs from mature *S. mansoni* can activate p38 MAPK and ERK in younger worms. **a** Male, or **b** female worms, obtained 33 days post-infection, were exposed to opposite sex ESPs derived from mature (45 day) or similar age (33-day) worms, or were not exposed to opposite sex ESPs (controls), for 15 min. Worm proteins were extracted, and equal protein amounts processed for western blotting with anti-phospho-p38 MAPK or -ERK antibodies. Blots were re-probed for actin as loading control. Vertical dotted lines on the blot images indicate non-adjacent lanes. Mean relative change in phosphorylation (±S.E.M.; n = 3 biological replicates) in worms was calculated, against control, unexposed, values (assigned a value of 1, dotted line) based on band intensity analysis after normalisation against actin; *p ≤ 0.05, **p ≤ 0.01, and ***p ≤ 0.001 (ANOVA), when compared to control values.

When *S. mansoni* become fully mature and commence egg laying they preferentially migrate to the mesenteric vasculature around the intestines^4^. We therefore tested whether ESPs released from D45 adult worms could affect protein kinase signalling in younger worms which typically reside within the blood vessel branches of the hepatic portal vein. Worms were collected 33 days post-infection (D33); upon perfusion the parasites were noticeably smaller in size than D45 worms and were underdeveloped. Exposure of the D33 male worms to ESPs from mature D45 females caused significant activation of both p38 MAPK and ERK at 15 min (Fig. 2a), in a similar fashion to that seen with mature worms (Fig. 1); activation also occurred following exposure of D33 females to mature D45 male worm ESPs (Fig. 2b). Interestingly, when ESPs were switched between the D33 male and female worms, these pathways were also activated, despite the immature status of the parasite (Fig. 2). These data suggest that ESPs released from fully mature male and female worms can signal to younger worms occupying a distinct vascular site; such communication might be relevant to parasite behaviour in vivo.

### Adult worm ESPs bind to the tegument of opposite sex adult worms

We next aimed to demonstrate, directly, the binding of ESPs derived from one sex to the tegument of the opposite sex using biotinylated ESPs. To enable this, it was necessary to: (i) pool ESPs from several worm batches, which involved cycles of freezing and thawing, and (ii) concentrate ESPs to a sufficiently small volume for the biotinylation reaction to occur. Importantly, neither freeze-thaw of ESPs nor concentration of ESPs using a 3000 Da MWCO filter (Supplementary Fig. 4) limited their ability to activate p38 MAPK or ERK in opposite sex adult worms.

**Fig. 3:**
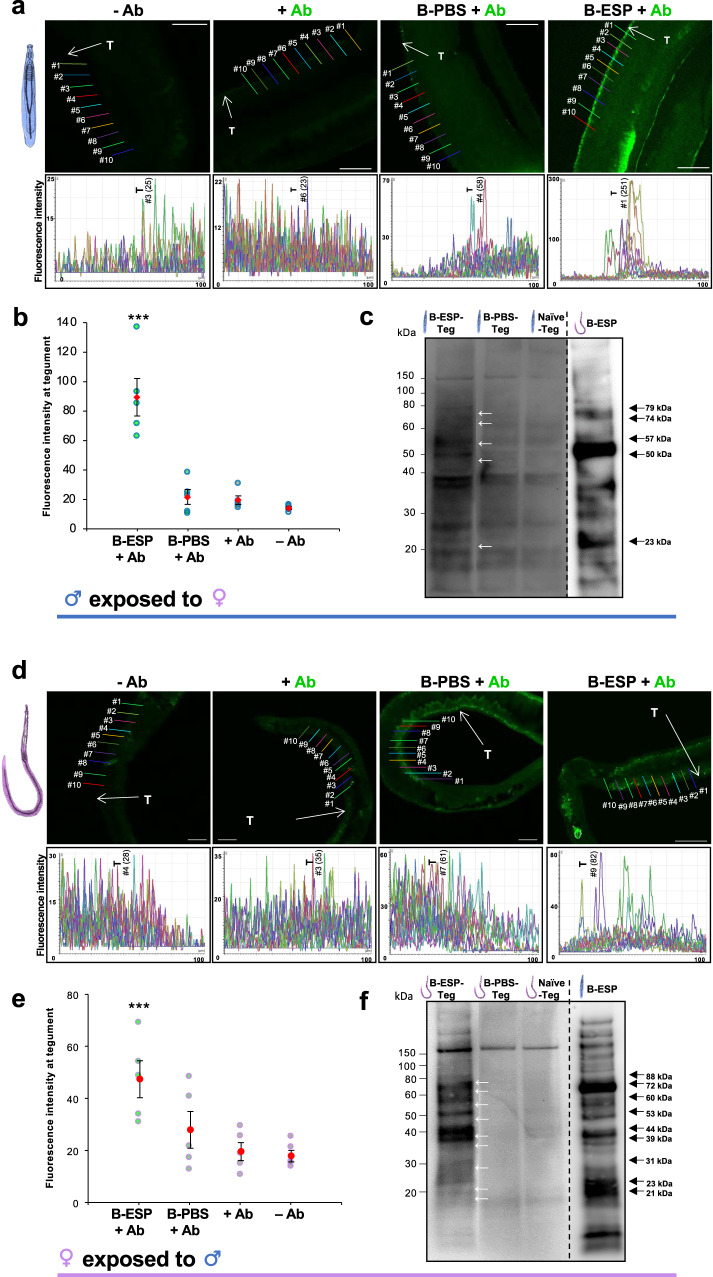
Adult *S. mansoni* ESPs bind to the surface of opposite sex worms. **a**–**c** Male, or **d**–**f** female worms, were exposed to biotinylated ESPs (B-ESP) derived from opposite sex adult worms, or PBS (B-PBS) control, for 15 min. **a**, **b**, **d**, **e** Worms, or **c**, **f** their corresponding tegument fractions (Teg), were then analysed by either CLSM or blotting, using anti-biotin FITC antibodies (Ab) and streptavidin-HRP conjugate, respectively. Quantitative analysis of fluorescence intensity at the tegument by CLSM was done for controls (-Ab, +Ab, B-PBS + Ab) and ESP-exposed (B-ESP + Ab) at ten random points for each worm, but at similar physical locations for each treatment to derive mean fluorescence intensities (±S.E.M.; n = 5 worms; bar graphs, ***p ≤ 0.001, compared to B-PBS controls); scale bar = 50 μm. For example, the tegument (T) fluorescence value (arbitrary units) for line ♯1 in the male B-ESP treatment is 251. The Y-axis of the Fluorescence Intensity plots are displayed in different scales for the tested conditions. For blotting, the approximate MWs of biotinylated ESP proteins that bound to the opposite sex worm teguments (B-ESP-Teg; white arrows) and were also present in the biotinylated ESPs (B-ESP; black arrows), but not robustly evident in the naïve tegument (Naïve-Teg) or control tegument (B-PBS-Teg) fractions are indicated.

Next, live worms were exposed to biotinylated ESPs, fixed (but not permeabilized), incubated in anti-biotin FITC antibodies, and visualised by confocal laser scanning microscopy (CLSM). Control worms were either untreated, incubated in anti-biotin FITC antibodies, or were exposed to mock biotin-treated PBS. Male adult worm autofluorescence was negligible, but some anti-biotin FITC signal was evident, presumably because certain proteins at the tegument surface may possess endogenous biotin (Fig. 3a). Male worms incubated in biotin-treated PBS (to control for labelling specificity and the removal of free biotin from ESP samples after biotinylation), followed by anti-biotin-FITC antibodies, also showed minimal staining (Fig. 3a). In contrast, male worms incubated with biotin tagged ESPs from adult females displayed striking fluorescence over their entire surface (Fig. 3a), including at the tubercles and the canyons between the tubercles (Supplementary Fig. 5). Significant binding was also seen at the tegument of female worms exposed to biotinylated male ESPs, although the difference in fluorescence signal between exposures and controls was less prominent (Fig. 3d). Quantification of fluorescence intensity revealed significantly greater fluorescence at the surface of adult male and female worms when exposed to biotinylated ESPs from the opposite sex (p ≤ 0.001), when compared with all control groups (Fig. 3b, e).

To further explore the nature of the adult worm ESPs, live male and female worms were exposed to opposite sex biotinylated ESPs and their teguments stripped for analysis by blotting. Control worms were exposed to biotin treated ‘RPMI’ (essentially PBS after concentration washes), and naive teguments were also stripped and probed to reveal the presence of naturally occurring biotinylated proteins in the tegument of adult male (Fig. 3c) and female (Fig. 3f) worms. At least five detectable proteins (MW range ~23 kDa - ~79 kDa) present exclusively in the biotinylated female worm ESPs bound the tegument of male worms (Fig. 3c), and at least nine male ESP proteins (MW range ~21 kDa - ~88 kDa) bound the surface of females (Fig. 3f). A heat treatment of the ESPs at 95 °C for 20 min to degrade any protein structures, did not prevent them from activating the kinases in opposite sex worms (Supplementary Fig. 6).

### Adult worms exposed to opposite sex ESPs display activated protein kinase signalling in the tegument, musculature, and reproductive organs

**Fig. 4:**
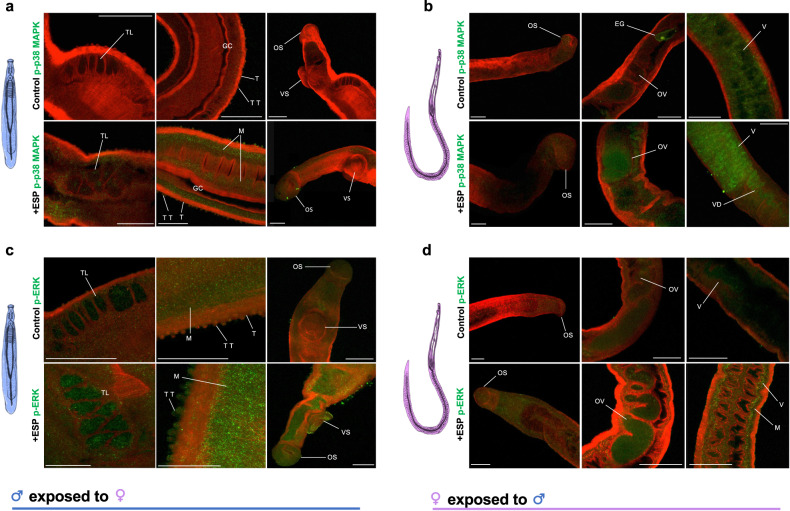
Adult worms exposed to ESPs from the opposite sex show activated protein kinase signalling in the tegument, musculature, and reproductive organs. **a**, **c** Adult male worms, or **b**, **d** adult female worms, were exposed to 24 h culture media containing ESPs from opposite sex adult worms for 15 min (or were not exposed, controls), fixed and processed for fluorescence microscopy by CLSM with **a**, **b** anti-phospho p38 MAPK, or **c**, **d** anti-phospho ERK antibodies, and Alexa Fluor 488 antibodies (green). Worms were also co-stained with rhodamine phalloidin (red) to reveal filamentous actin. Laser power and gain were adjusted to reveal low background for negative controls (Supplementary Fig. 7) and settings retained for imaging of all treated groups. Maximum projections of z-series from representative individual worms are depicted. EG, egg (in uterus); GC, gynaecophoral canal; M, muscle; OS, oral sucker; OV, ovary; T, tegument; TL, testicular lobes; TT, tubercles of the tegument; V, vitelline region; VD, vitelline duct; VS, ventral sucker. Scale bar = 50 μm.

Our approaches to functionally map phosphorylated protein kinases in intact *S. mansoni* have enabled us to identify cell signalling in situ^17,27–29,32^. We therefore functionally mapped phosphorylated p38 MAPK and ERK in adult worms to spatially determine kinase activation before and after exposure to opposite sex ESPs. Negative control worms displayed negligible fluorescence (Supplementary Fig. 7). Without ESP exposure, relatively low levels of p38 MAPK activation were observed in both sexes, but some staining was apparent in regions including in the female vitellaria (Fig. 4). However, following a 15 min-exposure to ESPs, activated p38 MAPK appeared prominent in the muscle layers, testicular lobes, and the tegument/tubercles of adult male worms (Fig. 4a), and in the ovaries and vitellaria of females (Fig. 4b). For ERK, activation in unexposed worms was evident in the tegument, muscle layers and testicular lobes of adult males, and the ovaries and vitellaria region of females (Fig. 4c, d), as previously described^30^. However, following a 15 min-exposure to opposite sex ESPs an apparent and sometimes striking activation of ERK was observed at the tegument, ventral sucker, musculature, and testicular lobes of males (Fig. 4c), with apparent activation also in the ovaries, vitellaria, tegument and musculature of females (Fig. 4d).

**Fig. 5:**
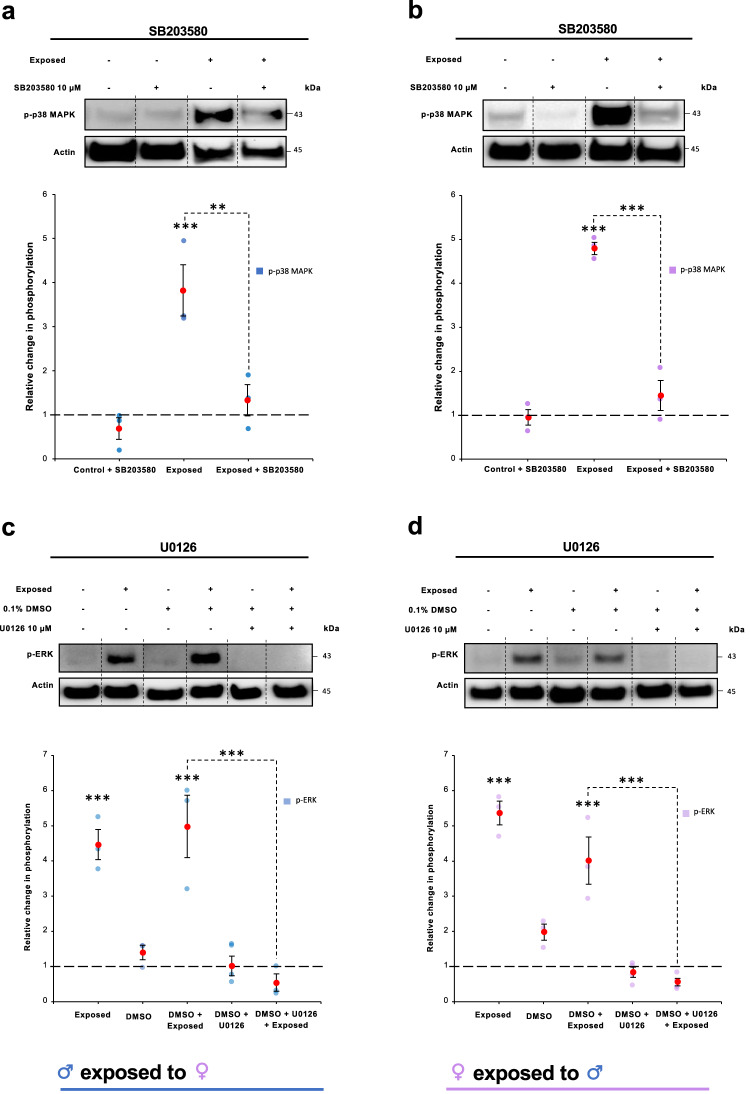
ESPs from adult *S. mansoni* activate p38 MAPK and ERK in the tegument of opposite sex worms and activation can be attenuated by SB203580 and U0126, respectively. **a**, **c** Adult male worms, or **b**, **d** adult female worms, were incubated for 1 h in (**a**, **b**) SB203580 (10 μM), or (**c**, **d**) U0126 (10 μM), or DMSO (vehicle for U0126), before being exposed to 24 h culture media containing ESPs (or not, control) from opposite sex adult worms for 15 min. Worm proteins were extracted and processed for western blotting with anti-phospho- p38 MAPK or -ERK antibodies. Blots were re-probed for actin as loading control. Vertical dotted lines on the blot images indicate non-adjacent lanes. Mean relative change in phosphorylation (±S.E.M.; n≥3 biological replicates) in worms was calculated, against control, unexposed and uninhibited, values (assigned a value of 1, dotted line) based on band intensity analysis after normalisation against actin; **p ≤ 0.01, and ***p ≤ 0.001 (ANOVA), when compared to control values, or between ESP exposed and inhibited (as shown).

Because (i) ESPs bound the tegument of opposite sex worms (Fig. 3); (ii) signalling responses to ESPs were observed in the tegument and underlying organs by immunofluorescence (Fig. 4); and (iii) the tegument is likely the major interface for signalling between the sexes, we next decided to expose live worms to opposite sex ESPs and strip teguments for analysis. In an attempt to block ESP-mediated signalling in the tegument, prior to ESP exposure, live worms were incubated in p38 MAPK and ERK inhibitors, SB203580 and U0126, respectively. We have previously shown that these inhibitors can block p38 MAPK and ERK activation in *S. mansoni*^27,28,30,33^. Robust ESP-mediated activation of both p38 MAPK and ERK was observed in tegument extracts of both male and female worms following ESP exposure (Fig. 5), in broad agreement with the confocal analysis (Fig. 4). Furthermore, the ESP induced activation of p38 MAPK was significantly attenuated by SB203580, with mean ~89% (p ≤ 0.01) and 88% (p ≤ 0.001) reductions in males and females, respectively; U0126 inhibited the ESP-mediated ERK activation to below basal levels in both sexes (Fig. 5).

Collectively these data reveal that ESP exposure results in p38 MAPK and ERK activation in the tegument of opposite sex worms, and that such ESP-mediated signalling possibly drives concomitant p38 MAPK and ERK activation in muscle and reproductive tissues.

### Exposure of adult worms to opposite sex ESPs evokes p38 MAPK- and ERK-dependent hyperkinesia

**Fig. 6:**
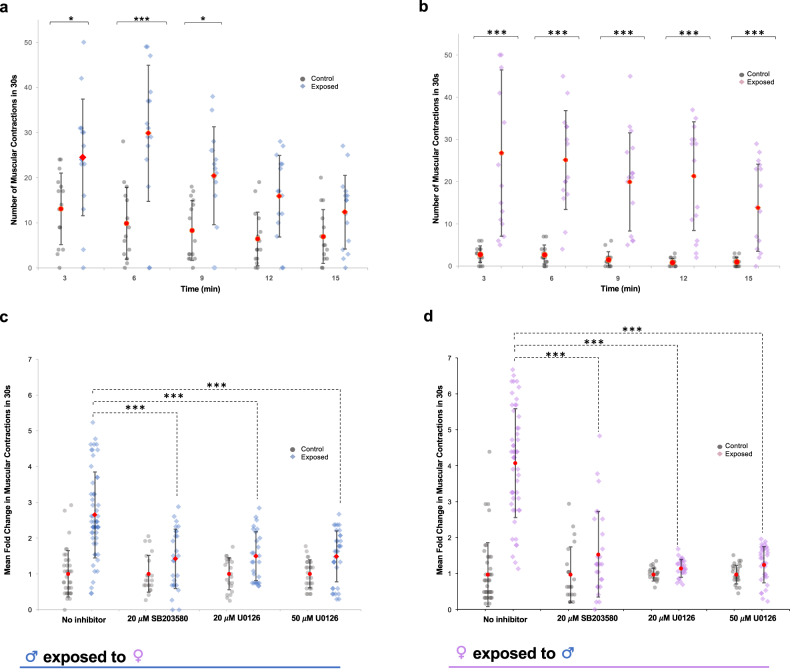
Adult *S. mansoni* ESPs induce hyperkinesia in opposite sex adult worms in a p38 MAPK- and ERK-dependent manner. **a** Adult male worms, or **b** adult female worms, cultured for 24 h were exposed to media containing ESPs from opposite sex adult worms or left untreated, and 30 s movies were recorded at indicated time points to evaluate the number of worm gross muscular contractions within 30 s; individual and mean values are shown (±S.D.; n = 15 worms per treatment/time from at least two independent experiments). **c** Adult male, or **d** adult female worms were incubated in 20 μM SB203580, 20 μM U0126, or 50 μM U0126, for 1 h prior to ESP exposure or left untreated (no exposure) with 30 s movies captured at 3 min. The fold change in contractions in response to ESP exposure was calculated for each treatment. *p ≤ 0.05, and ***p ≤ 0.001 (ANOVA), when compared to non-exposed control values or non-inhibited but ESP-exposed worms as shown. Representative movies are shown in Supplementary Movie 1.

Given that p38 MAPK and ERK were activated in the worm musculature following ESP exposure, we next considered that ESP interactions between worms might modulate movement. We therefore exposed adult worms to ESPs from the opposite sex and assessed their effects on worm motility. Remarkably, increased worm movement was detected for both sexes as early as 3 min (p ≤ 0.05) after ESP exposure (Fig. 6; Supplementary Movie 1). After 6 min exposure, male worms displayed a ~ 3-fold increase in movement (p ≤ 0.001; Fig. 6a), with female movements increasing ~9-fold (p ≤ 0.001; Fig. 6b); hyperkinesia was also observed in male worms for up to 9 min (Fig. 6a; p ≤ 0.05), whereas in females hyperkinesia was more pronounced and prolonged (Fig. 6b; p ≤ 0.001). Furthermore, while incubation of adult worms with 20 μM SB203580 or U0126 for 1 h did not suppress basal worm movement, treatment with 20 μM U1026 enhanced the movement of female worms (Supplementary Fig. 8). Nevertheless, 20 μM SB203580 or 50 μM U0126 appeared to significantly attenuate the ESP-mediated hyperkinesia in both sexes, by ~75% and ~70% in males (p ≤ 0.001; Fig. 6c) and 86% and 93% in females (p ≤ 0.001; Fig. 6d), respectively (Supplementary Movie 1). These data suggest a functional role for the ESPs in modulating opposite-sex adult worm motility and support a tentative role of p38 MAPK and ERK pathways in mediating this response.

### Exposure of adult worms to opposite sex ESPs stimulates p38 MAPK- and ERK-dependent stem cell proliferation

**Fig. 7:**
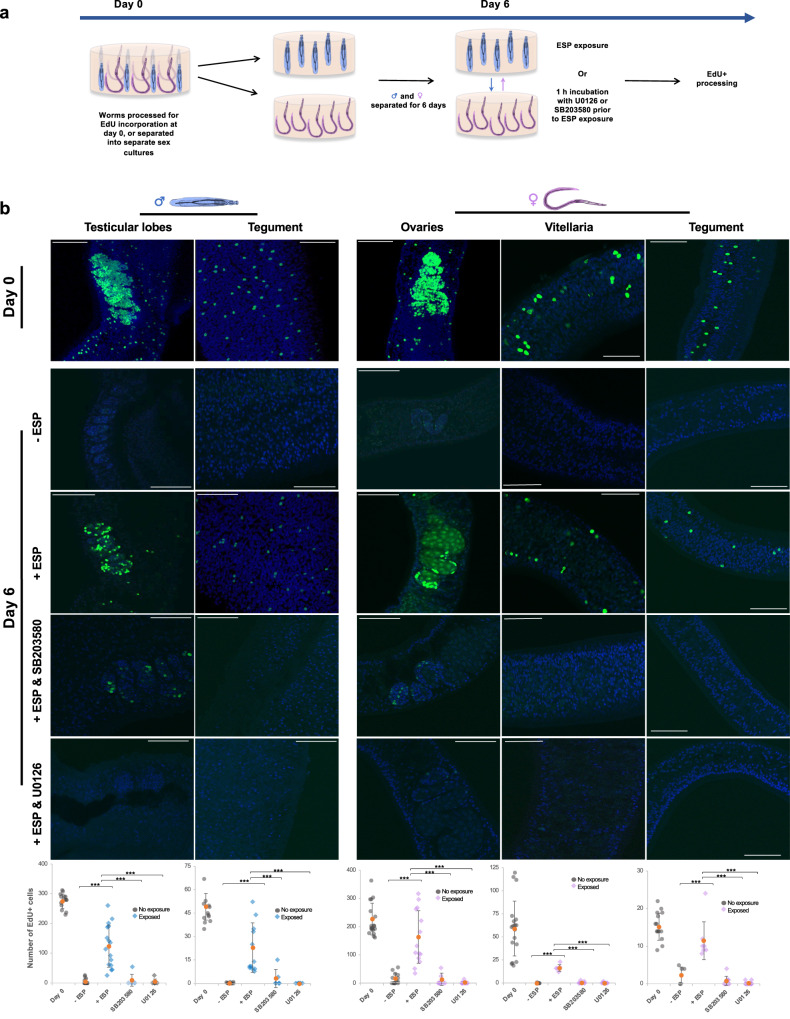
Adult *S. mansoni* ESPs rescue stem cell proliferation in adult worms from single sex cultures in a p38 MAPK and ERK-dependent manner. **a** Cartoon illustrating the experimental workflow. **b** Adult male or female worms were either chased with EdU on day 0 for 24 h, or separated into groups of five males or females for 6 days prior to being exposed to opposite sex ESPs (or not, control) for 24 h either with or without 10 μM SB203580 or 10 μM U0126, and chased simultaneously with EdU for 24 h. Worms were then processed for EdU detection, stained with DAPI and images captured by CLSM. Representative images show maximum projections (50, 25, and 20 z-sections for the testes and ovaries, vitellaria, and teguments, respectively). Scale bar = 50 μm. Graphs show EdU^+^ cell counts (from individual sections of an entire z-stack) from at least three independent assays for male testicular lobes and tegument, and female ovaries, vitellaria and tegument with the mean (±S.D.) also shown; ***p ≤ 0.001 (ANOVA).

Given (i) the critical role of stem cells in schistosome development, reproduction^13^ and tegument maintenance^15^, and (ii) the apparent activation of p38 MAPK and ERK in the reproductive organs and tegument, after opposite sex ESP exposure (Figs. 4, 5), we hypothesised that ESPs from one sex might modulate stem cell proliferation in the other sex. Separate groups of adult worms were therefore chased with EdU on (i) day 0 to determine baseline cell proliferation; and (ii) after six days in homosexual culture, with and without exposure to opposite sex ESPs (Fig. 7a). We reasoned that stem cell proliferation might diminish in vitro after several days to a level where it could then be stimulated. Control worms not chased with EdU displayed minimal fluorescence (Supplementary Fig. 9). Day 0 EdU-chased worms possessed high numbers of proliferating EdU^+^ cells in the testicular lobes of males, the ovaries and vitellaria of females, and the sub-tegument of both sexes (Fig. 7b). However, after 6 days culture in single sex groups, the number of EdU^+^ cells in both male and female worms plummeted, with a ≥98% decrease within the male testicular lobes and sub-tegument and a 93%, 99% and 85% decrease in the female ovaries, vitellaria, and sub-tegument, respectively (p ≤ 0.001). Strikingly, however, when male worms were exposed to female ESPs, the number of EdU^+^ cells increased substantially in the testicular lobes (25-fold increase) and sub-tegument (70-fold) (Fig. 7b; p ≤ 0.001). Furthermore, when these worms were incubated with SB203580 or U0126 for 1 h prior to exposure to female ESPs and EdU chase, the increase in proliferating cells in these organs was abolished (p ≤ 0.001; Fig. 7b). Similar effects were observed in female worms exposed to male ESPs, with ESP-mediated increases in EdU^+^ cell number observed in the ovaries (10-fold), vitellaria (60-fold) and sub-tegument (5-fold) (Fig. 7b; p ≤ 0.001); this stimulation was completely attenuated when female worms were pre-incubated with SB203580 or U0126 prior to exposure to male ESPs (p ≤ 0.001; Fig. 7b). Analysis of publicly available single cell RNA-seq data from mature adult *S. mansoni*^34^ revealed that p38 MAPK and ERK are robustly expressed in the proliferative neoblast cells, germinal stem cells, and S1 cells of the vitellaria in male and female adult worms; they are also expressed in the progeny of these stem cell types and the differentiated resultant cells (early TSP-2 + , late male or female germinal stem cells, and early/late vitellocytes) (Supplementary Fig 10). Collectively our data support the model that schistosome ESPs can modulate opposite sex stem cell proliferation and that p38 MAPK and ERK may be involved in this process.

## Discussion

**Fig. 8:**
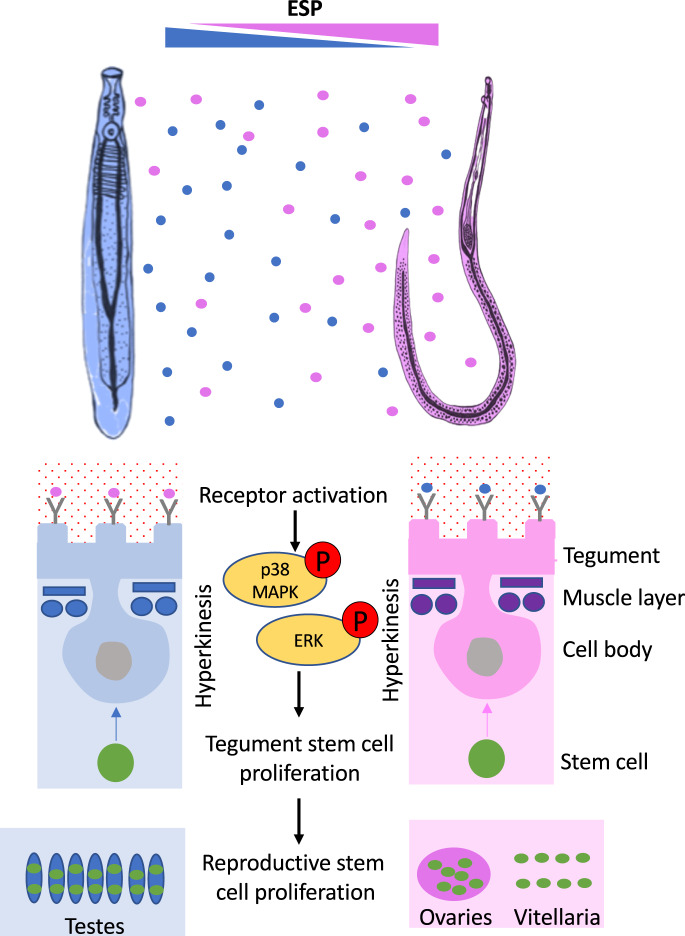
Model for ESP-mediated p38 MAPK and ERK activation in *S. mansoni* and downstream induction of hyperkinesia and stem cell proliferation. The ESPs released by either male or female worms are sensed by opposite sex worms through the binding of ESPs to receptors at the tegument surface. Tegumental p38 MAPK and ERK are activated *via* phosphorylation in response to receptor activation, and co-ordinate the proliferation of somatic stem cells that may induce tegument growth and tissue regeneration. These pathways, also activated in the muscle cells, would promote hyperkinesia. Finally, p38 MAPK and ERK signalling stimulate the proliferation of the germinal stem cells in the testes and the ovaries, and stem cells in the vitellaria, possibly supporting the maturation and reproduction of both sexes.

Dioecy implies an interplay between opposite sexes; however, the nature of such interactions in schistosomes remains to be extensively explored at the molecular level. The findings reported here support for the first time the hypothesis that soluble ESPs released from adult worms can directly interact with the surface of opposite sex worms and drive hyperkinesia and somatic/germinal stem cell proliferation *via* p38 MAPK and ERK signalling pathways. Because these responses are contact-independent, ESPs could potentially offer a remarkable mechanism for intersex communication and signalling over distance within the vasculature that have not, to the best of our knowledge, been previously studied (see model in Fig. 8). This mechanism would complement the one triggered by physical interactions between male and female parasites recently reported^24^.

We discovered that p38 MAPK and ERK are activated in male or female worms upon encountering ESPs from the opposite sex, with effects observed following exposures between mature (D45) worms, D33 worms, and between D45 and D33 worms. However, the activation of two other protein kinases investigated, PKA and PKC, was unaffected. P38 MAPK and ERK were activated (*via* phosphorylation) in response to the opposite sex ESPs, predominantly in the tegument/sub-tegument, muscle, and gonads. Given that the ESP-mediated hyperkinesia and stem cell proliferation were blocked by p38 MAPK and ERK inhibitors, it is tempting to speculate that ESPs are sensed at the parasite surface and signals triggered and propagated *via* these pathways across the tegument syncytium. Given that schistosomes lack a circulatory system, a question remains as to how signals received at the tegument could affect internal organs, such as the gonads? The nervous system could facilitate this signalling, as the peripheral nervous system extends to the worm surface, and it innervates the schistosome organs^29,35^. Notably, ESPs appeared to bind to the male worm tubercles, locations that are known to possess sensory papillae^36^, and which have been shown by us and others to act as a signalling interface^29,37^.

In terms of ERK and p38 MAPK signalling in schistosomes, we have previously shown that human EGF and insulin stimulate ERK phosphorylation in *S. mansoni* schistosomules^33^, that the ERK inhibitor U0126 reduces adult worm oviposition^30^, and that the p38 MAPK inhibitor SB203580 suppresses the transition from miracidia to mother sporocysts^26^. More recently, RNAi-mediated knockdown of p38 MAPK in *S. mansoni* was found to blunt egg production, suppress ovary development, and cause tegument damage in adult worms^38^, while RNAi of ERK was associated with underdeveloped ovaries^39^. These findings support a prominent role for p38 MAPK and ERK pathways in germinal and somatic stem cell proliferation, as observed in the current study. Indeed, it is well established that the ERK and p38 MAPK pathways play multiple roles in diverse organisms^40^, in particular in cell proliferation and differentiation^41^ and mammalian stem cell regulation^42^. Interrogation of publicly-available RNA-seq data derived from single cells of mature adult *S. mansoni*^34,43^ (Supplementary Fig. 10) demonstrated that proteins predicted to be associated with p38 MAPK and ERK in the parasite, including those acting upstream (e.g. growth factor receptors and the hierarchical kinases, MAPKK and MAPKKK), or downstream (e.g. transcription factors) of the kinase are robustly expressed in both somatic and germinal stem cells and their progeny (Supplementary Fig. 10). These findings support that p38 MAPK and ERK pathways are intact in both types of stem cells. Elucidation of the precise mechanisms by which p38 MAPK and ERK drive stem cell proliferation in the parasite is now warranted, particularly as certain molecular details may differ from the dogma established for higher organisms^40–42^.

The effect of p38 MAPK and ERK on worm motility is supported by the broad roles assigned to the MAPKs^40^ in smooth muscle contraction^44–46^. Indeed, motility of the nematode worm *Caenorhabditis elegans* has been shown to be ERK dependent^47^. Hyperkinesia and stem cell proliferation, induced in both male and female worms in response to ESPs from the opposite sex, may underly the success of *Schistosoma* mating. Firstly, mutual chemoattraction between opposite sexes of adult *S. mansoni* has previously been reported^48,49^, although some studies suggest that chemoattraction mainly occurs in females responding to male-released factors^50^. Nevertheless, a heterosexual hypermotile parasite response to ESPs could enhance the opportunity of male and female worms moving closer to one another before pairing. Although, in vivo, ESPs would be diluted in the blood, it is plausible that an ESP gradient could be sensed, particularly within the portal system where the worms no longer circulate and dwell within the mesenteric veins^4^. Secondly, pairing between schistosomes is crucial for sexual maturation, particularly of the female worm^19–21^. However, given that successful egg production and zygote formation by females depends on the development of: (i) oocytes in the ovary; (ii) S1 cells into S4 cells in the vitellaria; and (iii) sperm in male testicular lobes^11^, it seems that the ESP-mediated germinal stem cell proliferation may offer a previously uncharacterised molecular mechanism involved in the maturation and reproduction of both sexes. Thirdly, the somatic stem cells of schistosomes mirror neoblast cells in their free-living relatives, the planarians^51^. Throughout the development of *S. mansoni*, these cells differentiate into somatic tissues including muscle, tegument, and gut, playing a key part in the rapid growth and maintenance of the parasite inside the hostile environment of the host^13,51^. It has been demonstrated that the neoblasts play a critical role in supplying new cells to the tegument^15,52^, being particularly relevant for tegument turnover^16^, a process often related to host immune evasion. Thus, the finding that opposite sex ESPs stimulate stem cell proliferation in the schistosome tegument opens opportunities to investigate further the benefits of heterosexual worm interactions on the maintenance and integrity of the schistosome tegument and worm longevity.

While we have studied the involvement of individual protein kinases, it is important to highlight that protein kinases rarely work independently and that p38 MAPK and ERK have upstream regulators and downstream targets^40^, which include proteins that facilitate crosstalk with other pathways. Interrogation of the STRING protein-protein association database^53^ revealed 33 and 38 putative high-confidence (interaction score, 0.70) first shell interacting proteins for p38 MAPK (Smp_191040) and ERK (Smp_047900), respectively, in *S. mansoni* (Supplementary Fig. 10); 344 and 345 putative interacting proteins are revealed at medium (0.40) confidence. This highlights the complexity of p38 MAPK and ERK signalling in these worms, as in other organisms^40^. Furthermore, global phosphorylation changes seen in the adult worms following ESP exposure, identified using pS/pT/pY antibodies, also point to other proteins being phosphorylated in the worms by opposite sex ESP exposure, with differential patterns of phosphorylation seen between the sexes. Thus, it is probable that other protein kinase pathways are activated in male and female worms by ESPs, which in turn could drive functional responses. Further studies will follow to quantitatively determine the phosphoproteome^54^ and reveal additional pathways that are altered in male and female worms following ESP exposure. Collectively these strategies would offer a more global view of the impacts of ESPs on the developmental biology of male and female schistosomes.

Similar responses - p38 MAPK and ERK activation, hyperkinesia and stem cell proliferation - occurred in both sexes when exposed to ESPs from the opposite sex. However, homosexual ESP exposures did not induce signalling between worm groups. We speculate that male worms release a different repertoire of ESPs to the female worms, and this is somewhat supported by the banding pattern of the biotinylated ESPs. However, while these ESPs might bind different cognate receptors on the surface of each sex, coupling of pathways and possible redundancy amongst them could result in similar outcomes in terms of p38 MAPK and ERK signalling between the sexes. Moreover, schistosomes have evolved from hermaphroditic free-living planarian flatworms and are unique amongst the Platyhelminthes for being dioecious. Given that p38 MAPK and ERK seem to be critical for stem cell proliferation and motility in schistosomes, it is likely that such coupling of MAPK signalling to function would have been retained through evolution of dioecy, while divergent evolution may have occurred for ESPs and their cognate receptors. In this context, ERK has been shown to be important to regulating blastema cell differentiation during planarian regeneration^55^. It is important to also highlight that the protein kinase signalling responses observed might be due to multiple ESPs binding the surface of the worms rather than just one, and that downstream functional responses may extend beyond those characterised in the current work.

Crude schistosome ESPs comprise many molecules including those from extracellular vesicles^56^. Although investigations of *S. mansoni* ESPs have mostly focused on larval and egg-derived ESPs^57^, a proteomic analysis of adult *Schistosoma bovis*^58^ and *Schistosoma japonicum*^59^ ESPs have revealed ~400 separate protein spots and 101 identified proteins, respectively. Moreover, a recent study identified ~1000 proteins in *S. mansoni* adult worm ESPs collected over 7 days, with 370 and 140 proteins more abundantly expressed in males than females, respectively^60^. Despite developing several different biochemical approaches, including the use of cleavable biotin and neutravidin beads, to capture ESPs from teguments following ESP exposure, and using several hundreds of adult worms, we were unable to identify ESP-specific proteins in tegument fractions. The stimulatory factor(s) in our ESP preparations are likely considerably larger than the recently identified ß-alanyl-tryptamine (~231 g/mol, i.e., 231 Da) produced by male worms in response to pairing that influences female maturation^24^, because ESPs that were concentrated and extensively washed through 3,000 Da MWCO filters retained their ability to activate ERK and p38 MAPK. Our attempts to purify and characterise the biotinylated ESPs that bound the worm teguments were hampered to some extent by endogenous biotinylated proteins being present at the tegument surface. While endogenous biotin has been previously demonstrated in *S. mansoni* tissues by transmission electron microscopy^61^, our fluorescence microscopy data also point to the presence of this vitamin at the surface of the adult worm. These findings have implications for biotinylation-based studies that aim at isolating *Schistosoma* surface proteins for proteomic analysis^62–64^. This raises the question of whether such proteins that were thought to be truly surface exposed might instead reside within the tegument. Further investigations to address this issue are needed. Overall, we surmise that the low quantity of protein from the ESP fractions that bound to the teguments, when coupled with limitations in mass spectrophotometry, limited our ability to identify the ESPs that specifically bound the surface of opposite sex worms.

While this work focuses on the intersex effect of soluble ESPs and their importance to somatic/germinal stem cell proliferation, the pairing dependent sexual maturation of female schistosomes also seems to be regulated by protein kinases, with many of these enzymes transcribed in a gonad-preferential and/or pairing-dependent manner^65^. In this context, ERK (*Smp_047900*) gene expression in male or female adult *S. mansoni*, or their testis and ovary, appears to be unaffected by worm coupling (fold change < 1.5), whereas p38 MAPK (*Smp_191040*) displays increased expression in the ovary of paired females when compared with unpaired females^66^. Some protein kinases may play more distinct roles; for example, Smp_198810, most similar to human MAPK15, has an almost exclusive testis-specific expression^65,66^ that is upregulated after pairing, highlighting a possible role in spermatogenesis. Although the activity of protein kinases is largely governed by phosphorylation, rather than expression, differences in expression such as seen with Smp_198810 are probably important to schistosome sexual biology and warrant further investigation. For example, it would be intriguing to investigate the effects of *S. mansoni* ESP exposure on the dynamics of protein kinase expression in worms of the opposite sex and their reproductive organs.

Despite revealing and characterising a previously undescribed molecular mechanism underlying inter-sex communication in schistosomes (Fig. 8), several questions remain unanswered. Firstly, it is not known whether the observed ESP-mediated responses occur in vivo. It was not possible to use human serum in the current investigation because its addition would influence the activation of the kinases in a way that would make it difficult to evaluate the ESP-mediated response per se. Based on our current findings, we propose that medium- to long-range signalling between worms could occur, but if this is not the case and in the presence of human serum, it should still be possible for the male and female ESPs to influence one another in a heterosexual proximal interaction, including during pairing within the hepatic portal system. Secondly, although we demonstrate opposite sex ESP-mediated kinase activation between mature and younger worms, we have not investigated effects of ESPs on young worm development/maturation. Thirdly, it is not known whether the ESP-mediated heterosexual signalling observed here occurs in other schistosome species; however, we predict that, while ESPs and receptors might differ between species, the apparent core importance of p38 MAPK and ERK signalling to stem cell differentiation would be conserved.

The exchange of soluble low molecular weight compounds, such as carbohydrates, lipids, amino acids and peptides, between schistosomes *in copula*, and the provision of hormonal and nutritive material by the male to the female has previously been suggested by many authors for at least six decades (reviewed by Basch^11^). By studying the effects of ESPs derived from homosexual cultures on opposite sex worms, we reveal that ESPs induce hyperkinesia and somatic/germinal stem cell proliferation through p38 MAPK and ERK signalling, in a manner independent of physical contact. Our findings therefore offer crucial insights into a novel molecular mechanism of male-female schistosome communication that is relevant to worm survival, development, maturation, and reproduction.

## Methods

### Parasites

Mature adult (45 day, D45) and juvenile (33 day, D33) *S. mansoni* (Puerto Rican strain) were obtained from BioGlabs (Professor Mike Doenhoff, University of Nottingham), or from the Wellcome Sanger Institute (Cambridge, UK) as approved by the University of Nottingham Ethical Review Committee under Home Office licence 40/3595, or the Animal Welfare and Ethical Review Body of the Wellcome Sanger Institute under UK Home Office project license P77E8A062 (Gabriel Rinaldi), respectively. Laboratory animal use was carried out within designated facilities regulated under the terms of UK Animals Act 1986, complying with all requirements. The worms were recovered by hepatic portal perfusion of mice (female Balb/c *Mus musculus*) infected (at six weeks old) with *S. mansoni* cercariae as described^67^, washed and incubated in a Petri dish containing pre-warmed serum-free RPMI (Gibco) and antibiotics/antimycotics (Sigma) at 37 °C/5% CO_2_ for 1-2 h. The D33 worms were unpaired. Mature male and female worms were gently separated if paired and were transferred to individual wells of Nunc 48-well untreated tissue culture plates (Thermo Fisher Scientific). Each well contained 5 single-sex worms in 500 μl RPMI with antibiotics/antimycotics (1%) that were incubated for 24 h at 37 °C and 5% CO_2_.

### Worm exposures

After the 24 h incubation period, ~450 μl of the media containing the ESPs of male worms was gently exchanged with that of the female worms and vice versa (Fig. 1a), ensuring that a thin layer of the original medium covered the worms in the wells. The worms were exposed to the opposite-sex ESPs for increasing durations (5, 15, 30 and 60 min) before being immediately transferred into microfuge tubes on ice containing 200 μl chilled RIPA buffer (Cell Signalling Technology (CST)) and 2 μl HALT protease/phosphatase inhibitor cocktail (Thermo Fisher Scientific). For 0 min exposures, the media was gently removed and replaced to simulate change of media, then worms were transferred to chilled tubes on ice containing RIPA buffer as above. In experiments where 15 min exposures were done, control worm media was gently removed and replaced, and worms left for 15 min before processing. Same sex exposures were also performed, whereby media containing ESPs from a group of 5 male or female worms were exchanged with that of a different group of 5 worms of the same sex. All worms were homogenised using a plastic microfuge pestle and mortar (Kimble-Chase) and processed for western blotting. For some experiments, indicated in Results section, worms were instead processed for immunocytochemistry, tegument stripping, or video analysis.

To investigate the effects of heating or freeze/thaw on the biological activity of the adult worm ESPs, media containing ESPs from the 24 h single-sex cultures were recovered from wells and either: (i) heated in microfuge tubes for 20 min at 95 °C before cooling and equilibration at 37 °C/5% CO_2_ for 2 h, or (ii) frozen at −80 °C, and defrosted and equilibrated (37 °C/5% CO_2_ for 2 h) when needed. The media containing ESPs were then exchanged into 24 h cultures containing worms of the opposite sex; exposures were performed for 15 min and worms processed for western blotting.

For biotinylation experiments (below), adult worm ESPs were concentrated. To ensure that the concentrated adult male and female worm ESPs (now in PBS) possessed similar stimulatory effects to non-concentrated ESPs in RPMI, male or female worms were exposed for 15 min to concentrated ESPs (an equivalent volume from 5 worms) or PBS (control), diluted in overnight-incubated RPMI (total volume 500 μl). Specific volumes were employed for the experiments as accurate protein quantification was not possible due to the low protein concentration in the ESPs. Worms were then processed for western blotting.

### Isolation of tegument fractions following inhibitor treatment and ESP exposure

Adult worms (typically 60 of each sex), cultured in groups of 5 for 24 h, were incubated in either 10 μM SB203580 (p38 MAPK inhibitor, Calbiochem), 10 μM U0126 (MEK/ERK inhibitor, CST), water or DMSO vehicle (for SB203580 and U0126, respectively) for 1 h. They were then exposed to media containing ESPs from adult worms of the opposite sex, and each sex pooled in separate microfuge tubes on ice containing 100 μl phosphate buffered saline (PBS) to generate sufficient tegument extract for analysis. To isolate the tegumental fraction^17,68^, tubes were plunged in liquid nitrogen for 10 min. Next the sample was thawed, placed on ice, washed gently with 1 ml ice-cold PBS, and 100 μl ice-cold PBS added with 2 μl HALT protease/phosphatase inhibitor. Worms were then pulse-vortexed 20 times for 1 s each, the liquid fractions collected, and the worm carcasses removed. The entire extract, which represents the cytosolic and particulate fractions, was processed for western blotting as p38 MAPK and ERK resided in both fractions.

### SDS-PAGE and western blotting

An LDS sample buffer (4x) (Invitrogen) was added to whole adult worm homogenates or tegument fractions. The samples were heated at 95 °C for 5 min, sonicated (30 s) and pulse centrifuged. Protein lysates (typically ~10 μg for adult worms; estimated *via* detergent compatible Bradford assay, Pierce) were electrophoresed using Bolt Bis-Tris Plus gels (with MES/SDS buffer; Life Technologies) and separated proteins semi-dry transferred to nitrocellulose. Blots were stained with Ponceau S to confirm homogenous transfer and were blocked for 1 h in bovine serum albumin (BSA). After washing in tween tris-buffered saline (TTBS), blots were incubated in either anti-phospho S/T/Y antibodies (Abcam, ♯SPM101), or anti-phospho -p38 MAPK (Thr^180^/Tyr^182^), -ERK (p42/p44 MAPK) (Thr^202^/Tyr^204^), -PKA-C (Thr^197^), or -PKC (pan) (ς Thr^410^) antibodies (CST, ♯9215, ♯9101, ♯4781, and ♯2060, respectively), each at 1:1000 in 1% BSA. Blots were then incubated in horseradish peroxidase (HRP)-conjugated anti-rabbit secondary antibodies (CST, 1/5000; 2 h) and bands visualised using ECL Prime (GE Healthcare) or West Pico (Thermo Fisher Scientific) chemiluminescent reagents and a GeneGnome (Syngene) chemiluminescent detection platform. To re-probe, blots were stripped with Restore Western Blot Stripping Buffer (Thermo Fisher Scientific); actin was detected with HRP-conjugated anti-actin antibodies (1:3000, 3 h; Santa Cruz Biotechnology, ♯sc-47778). Band intensities were quantified using GeneTools (Syngene) and phosphorylation levels normalised to actin band intensity.

### Functional mapping of protein kinase activation by immunofluorescence

Following ESP exposure, adult worms were fixed in ice-cold acetone for 30 min, washed twice in 1 ml PBS, blocked in 1% glycine for 15 min, washed again in PBS, and incubated in 0.3% triton X-100 for 1 h^29,30^. After a further PBS wash, worms were blocked in 10% goat serum for 1 h, washed in PBS and incubated in anti-phospho p38 MAPK (Thr^180^/Tyr^182^) or anti-phospho ERK (Thr^202^/Tyr^204^) antibodies (1:50 in 5% BSA) for 3 days with rotation. After extensive washing in PBS (3 × 30 min) worms were incubated in Alexa Fluor 488 anti-rabbit secondary antibodies (1:250 in 5% BSA) (Life Technologies)/rhodamine phalloidin (0.5 μg/μl; Sigma) for 2 days. Worms were finally washed thoroughly, sealed under coverslips in VectaShield anti-bleaching medium (Vector Laboratories) and visualised on a Leica SP2 AOBS CLSM with images captured and analysed using Leica software. Laser power and gain were adjusted to reveal low background for the negative controls and settings then retained for imaging of all worms.

### Biotinylation of adult worm ESPs

Frozen media containing ESPs from 24 h adult worm cultures (typically 5 ml from 50 worms for each sex) was thawed and concentrated using Vivaspin 6 ml spin columns, 3000 Da MWCO (Sartorius), in a centrifuge (swing bucket rotor; 4,000 x *g*) to ~500 μl. The filtrate was removed, and 4 ml PBS added to the Vivaspin column, which was centrifuged; this step was repeated 3 times to remove excess RPMI that could interfere with biotinylation. RPMI was processed in a similar way to serve as a negative control. The final ESP concentrate typically comprised 300 μl male or female ESPs in PBS. Next, biotin (EZ-link Sulfo-NHS-Biotin kit; Thermo Fisher Scientific) was dissolved, 1 mg in 200 ml PBS, and 20 μl biotin solution added to each sample or to the PBS control (derived from the RPMI control) and incubated on ice for 2 h. The Zeba desalting columns were pre-washed 3 times with PBS, biotinylated samples added together with 100 μl ultra-pure water and then centrifuged at 1000 x *g* for 2 min to remove unbound biotin. The final biotinylated samples were collected and frozen at −80 °C.

### Analysis of ESP binding by microscopy and blotting

Biotinylated adult male or female worm ESPs or PBS controls (100 μl of each) were transferred to individual wells of a 96-well tissue culture plate (Nunc) and worms from 24 h cultures carefully included (males with female ESPs and vice versa, plus PBS controls, 10 worms/well). Worms were exposed to the ESPs/PBS for 15 min then fixed immediately in 4% paraformaldehyde for 24 h at 4 °C. Worms were then washed twice with 1 ml PBS, 5 min each, and blocked with 1% BSA in PBS for 1 h. Next, worms were incubated in anti-biotin FITC antibodies (1:200 in 1% BSA; Sigma-Aldrich ♯F4024) for 2 h before washing 5 times with 1 ml PBS and mounting on slides with SlowFade Gold antifade mountant containing DAPI (Thermo Fisher Scientific). Control worms were prepared with the antibody but without ESP exposure, and with no treatment. Worms were visualized by CLSM to capture single z-scans through the body of each worm; laser power and gain were kept constant for imaging of all worms. Images collected were analysed using the Leica quantification tool by measuring the fluorescence intensity across the tegument of worms at ten randomly selected points, albeit at a similar physical location on each worm.

For blotting, 20 adult male worms were exposed to biotinylated female worm ESPs (a volume of concentrate equivalent to that from 20 worms) for 15 min; controls included 20 each of naïve worms and worms exposed to PBS. The same was done for the female worms but with male biotinylated ESPs. Worm teguments were stripped and processed as detailed above, except that the resultant fractions were further centrifuged at 12,000 x *g* for 30 min at 4 °C to isolate only the particulate, membranous fraction, which was then solubilised in RIPA buffer (CST). The tegument proteins were electrophoresed, blotted to nitrocellulose, and blots probed with streptavidin HRP-conjugate (Thermo Fisher) to identify biotinylated proteins. Approximate MWs of proteins were derived from graphs of log MW against band migration distance.

### Effect of ESPs and inhibitors on worm motility

Adult worms cultured for 24 h were exposed to ESPs from worms of the opposite sex and movies captured at 0, 3, 6, 9, 12, and 15 min after exposure, using a Moticam 1080 digital camera attached to a Motic SMZ171 stereomicroscope. For controls, media was gently removed and replaced to simulate media change and movies were captured. Gross muscular contractions/random movements within 30 s at each time point were counted. For filming, camera position, light intensity, frames per second (30) and magnification remained constant. In parallel experiments, worms were also incubated in 20 μM SB203580, and 20 μM or 50 μM U0126, for 1 h prior to exposure to opposite sex ESPs, or not, for 3 min to determine the effects of pathway inhibition on basal and ESP-induced motility.

### Effect of ESPs and inhibitors on stem cell proliferation

To investigate cell proliferation, Click-iT EdU with Alexa Fluor 488 azide (Thermo Fisher Scientific) was used according to described protocols^69^ and manufacturer’s instructions. Groups of 5 adult male or female worms (all worms of similar size) were cultured in RPMI and antibiotics/antimycotics for 6 days. Thereafter, media containing the worm ESPs were exchanged between the cultures to provide opposite sex exposures, 20 μM EdU immediately added, and worms cultured for a further 24 h. Positive control worms were EdU-chased on day 0 to assess ‘normal’ cell proliferation levels, and negative controls (no ESP exposure) were EdU-chased at the same time as the ESP treated worms. All worms were, however, processed at the same time and with the same reagent batches. After the EdU chase, worms were transferred to 200 μl PBST fixative (1 ml paraformaldehyde, 3 ml PBS, and 12 μl Triton X-100) for 4.5 h. The fixative was then removed, and worms dehydrated in 50% MeOH followed by 100% MeOH, before placing at −20 °C in 100% MeOH. The next day, worms were rehydrated with 50% MeOH for 10 min, washed with PBST, and treated with proteinase K (6 μg/ml in 29.5 mM Tris HCl, pH 8.0) for 25 min, before final PBST incubation for 1 h. Worms were next washed twice in 3% BSA in PBS and incubated in Alexa Fluor 488 reaction cocktail (100 μl reaction buffer, 100 μl buffer additive, 800 μl copper sulphate) for 3 h in the dark. Finally, worms were washed twice, 15 min each, in 3% BSA in PBS, and mounted under coverslips in SlowFade Gold mountant containing DAPI. In parallel experiments, worms were pre-incubated with 10 μM SB203580 or 10 μM U0126 for 1 h, on day 6 of treatment before exchange of media containing ESPs.

The mounted parasites were visualized using a CLSM. A series of single z-sections, z-stacks and maximum projections were obtained for male (tegument, testes) and female (tegument, ovaries and vitellaria) worms, and EdU^+^ cells counted; when no EdU^+^ cells were present, a zero was recorded and a single z-section captured. For testes and ovaries, EdU^+^ cells were counted throughout the whole organ. However, for the tegument and vitellaria, EdU^+^ cells were counted within the total area of three captured images for each worm: (i) male tegument, images captured were across the dorsal surface of the worm, proximal to the oral sucker, mid-region, and towards the worm posterior; (ii) female tegument, images captured were across the dorsal surface proximal to the ovaries, ventral and oral suckers to ensure that EdU^+^ cells were tegumental and not from the underlying vitellaria; and (iii) vitellaria, images captured were proximal, mid-way and distal to the ovaries. Laser power and gain were adjusted to reveal low background for negative controls and the same settings retained for imaging of all worms and worm regions.

### Additional data sources

Investigation of putative protein-protein interactions was undertaken using the STRING protein-protein association database (www.string-db.org)^53^ querying Smp_191040 and Smp_047900 identifiers for p38 MAPK and ERK, respectively. Cell type gene expression maps were generated for selected Smp identifiers using the SchistoCyte Atlas (www.collinslab.org/schistocyte/)^34^.

### Statistics and reproducibility

Statistical analysis of movement, stem cell proliferation and western blot densitometry data were performed with the assistance of Minitab (version 19). Biologically independent experiments were performed with the number for each investigation detailed in the figure legends. Differences between the means of groups were tested using one-way analysis of variance (ANOVA) followed by Fisher’s multiple comparison post-hoc test. Differences of p ≤ 0.05 were considered statistically significant.

### Reporting summary

Further information on research design is available in the Nature Portfolio Reporting Summary linked to this article.

### Supplementary information

Supplementary Figures

Description of Additional Supplementary Files

Supplementary Movie 1

Supplementary Data 1

Reporting Summary

## Data Availability

All data that have been generated or analysed during this study are included in the published article and the associated supplementary files. Where cropped western blot images have been used in the results figures, uncropped images have also been provided (Supplementary Figs. 11–18); raw data for graphs are presented in Supplementary Data 1.
