# Supplementary material for: Schistosoma mansoni excretory-secretory products induce protein kinase signalling, hyperkinesia, and stem cell proliferation in the opposite sex
Source: Commun Biol. 2023 Sep 26;6:985. doi: 10.1038/s42003-023-05333-9 (PMC10522684; doi:10.1038/s42003-023-05333-9)
Supplement: Supplementary file 1 — Supplementary Figures [file 42003_2023_5333_MOESM1_ESM.pdf]

Supplementary Figures

Schistosoma mansoni excretory secretory products induce protein kinase signalling, hyperkinesia, and stem cell proliferation in the opposite sex

Eman M.N. Shakir, Gabriel Rinaldi, Ruth S. Kirk, Anthony J. Walker

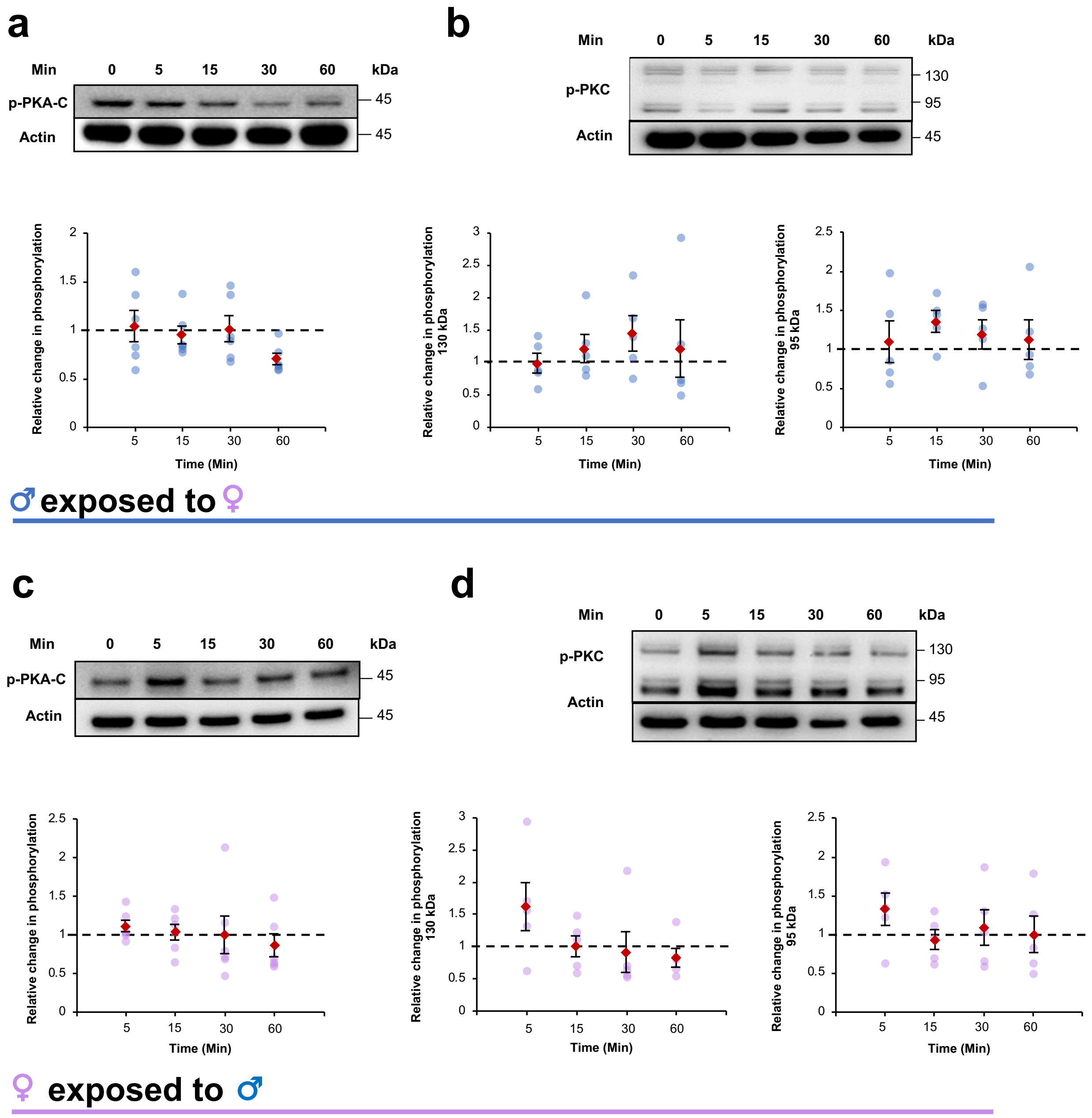

Supplementary Fig. 1 Adult *S. mansoni* ESPs do not induce PKA or PKC activation in opposite sex worms. a-b Adult male worms, or **c-d**, adult female worms, were exposed to 24 h culture media containing ESPs from opposite sex adult worms for increasing durations, worm proteins extracted, and equal protein amounts processed for western blotting with anti-phospho-PKA-C or -PKC antibodies, respectively. Blots were also probed for actin as loading control. Mean relative change in phosphorylation (±S.E.M.; n≥5 biological replicates) in worms over time was calculated (graphs), against control 0 min values (assigned a value of 1, dotted line), based on band intensity analysis after normalisation against actin.

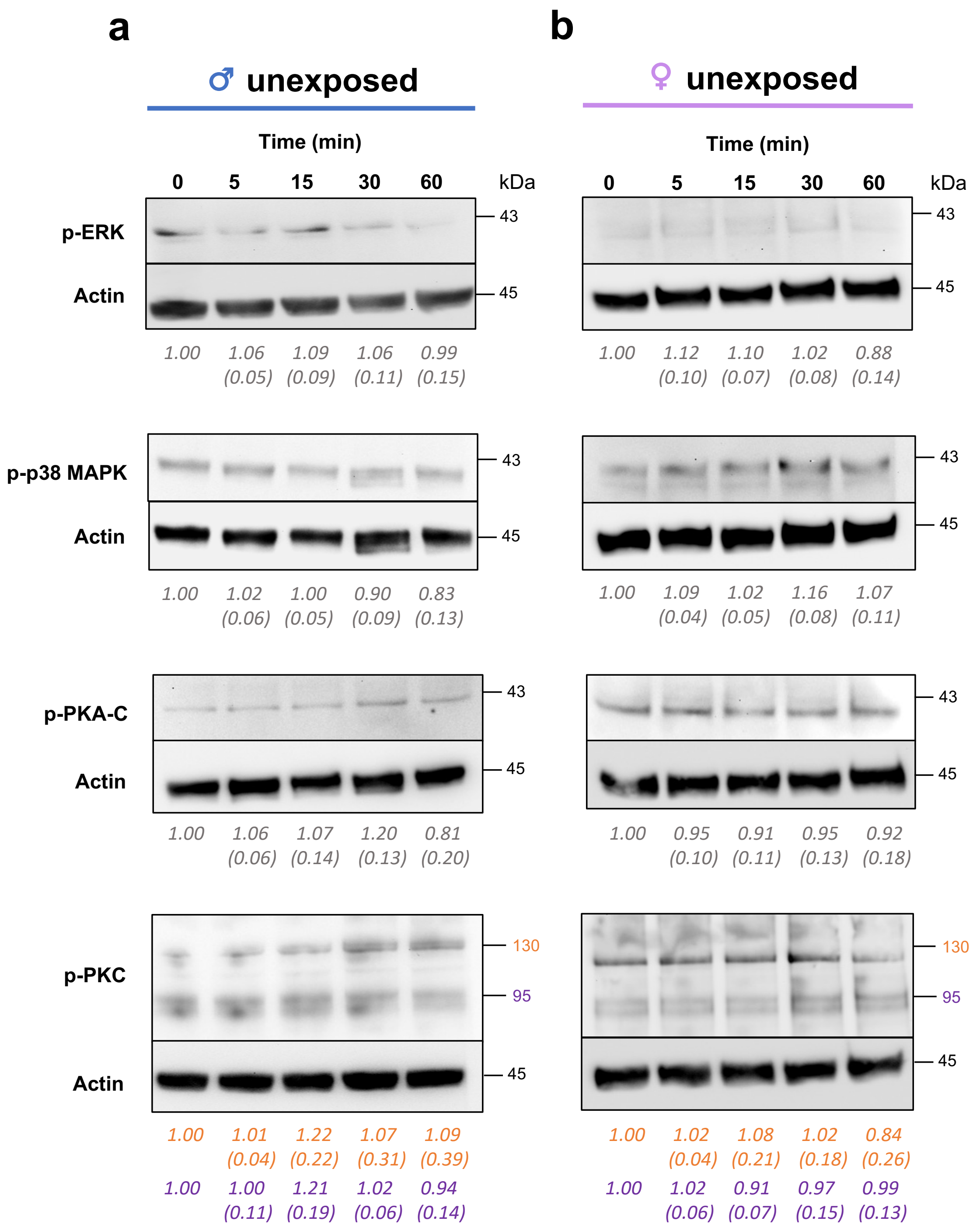

Supplementary Fig. 2 Protein kinase activation does not change in adult *S. mansoni* without ESP exposure. a Adult male, or **b** adult female, worms were left undisturbed in culture media for increasing durations, worm proteins extracted, and equal protein amounts processed for western blotting with anti-phospho-ERK, -p38 MAPK, -PKA-C, or -PKC antibodies. Blots were also re-probed for actin as loading control. Mean relative change in phosphorylation (values under lanes, ±S.E.M in brackets; n=3 biological replicates) in worms was calculated, against control (0 min) values (assigned a value of 1) based on band intensity analysis after normalisation against actin. The different colours used for p-PKC values represent the different PKCs present.

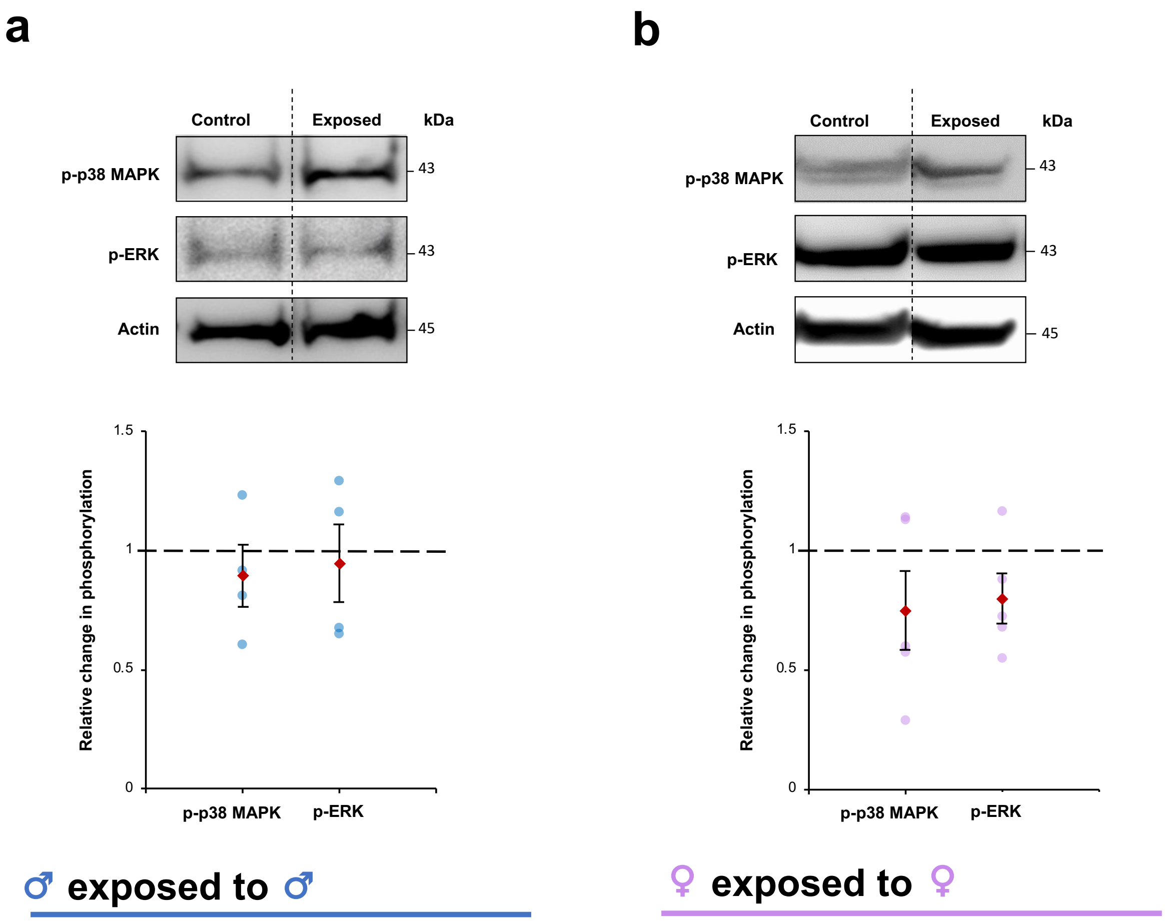

Supplementary Fig. 3 Same sex ESPs do not induce p38 MAPK or ERK activation in adult *S. mansoni*. a Adult male, or **b** adult female, worms were exposed to culture media containing ESPs from a different group of same sex adult worms or not (control) for 15 min, worm proteins extracted, and equal protein amounts processed for western blotting with anti-phospho-p38 MAPK or -ERK antibodies. Blots were also re-probed for actin as loading control. Vertical dotted lines on the blot images indicate non-adjacent lanes. Mean relative change in phosphorylation (±S.E.M.; n=4 biological replicates) in worms was calculated, against control, unexposed, values (assigned a value of 1, dotted line) based on band intensity analysis after normalisation against actin.

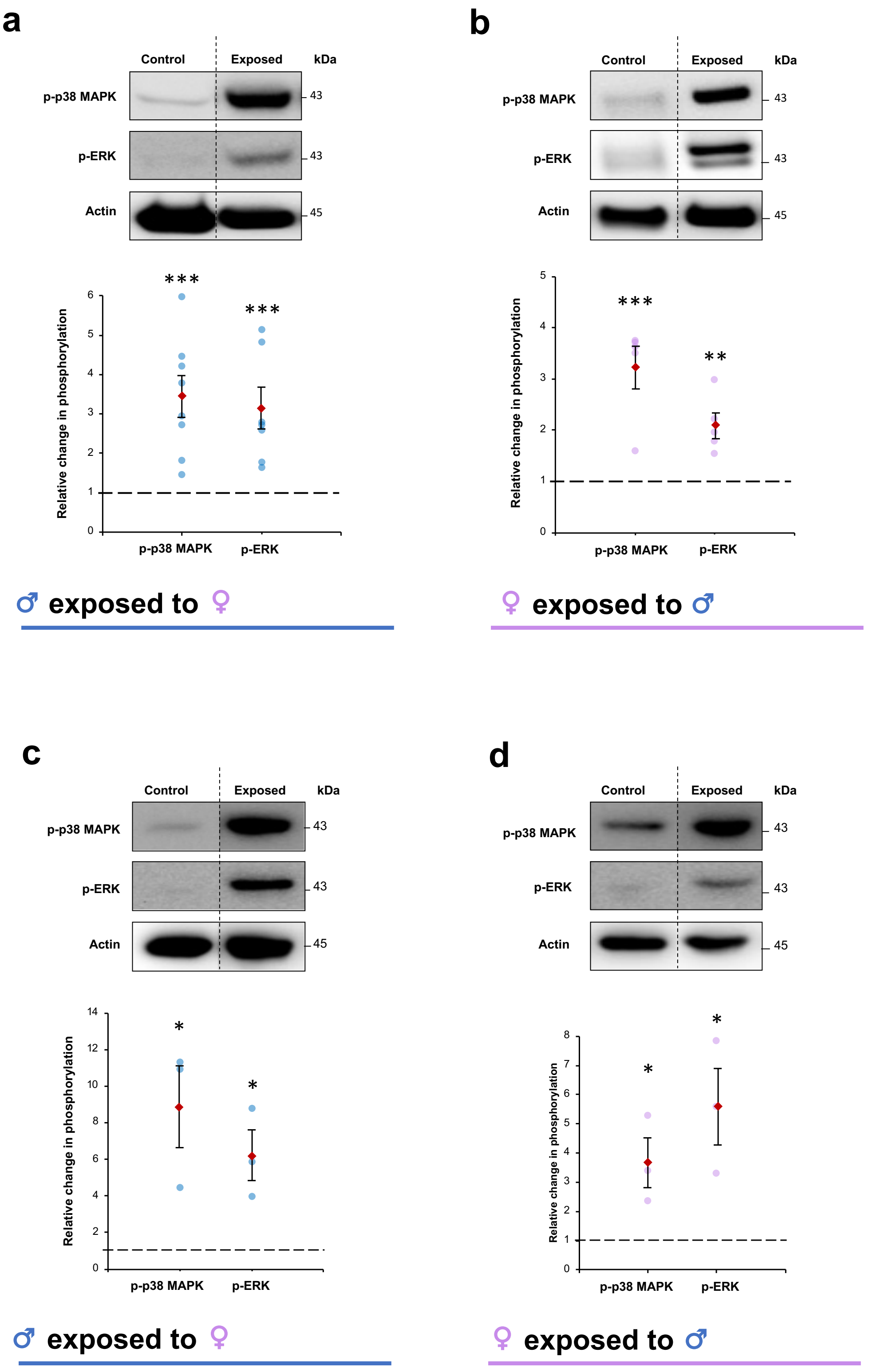

Supplementary Fig. 4 Neither freeze-thaw, nor concentration, of adult S. *mansoni* ESPs removes their ability to activate p38 MAPK or ERK in opposite sex worms. Worm culture media containing ESPs were either subject to **a-b** concentration through a 3000 Da MWCO filter, or **c-d** freeze-thaw. Worms were then exposed to the processed opposite sex ESPs or not (control) for 15 min, worm proteins extracted, and equal protein amounts processed for western blotting with anti-phospho-p38 MAPK or -ERK antibodies. Blots were also re-probed for actin as loading control. Vertical dotted lines on the blot images indicate non-adjacent lanes. Mean relative change in phosphorylation (±S.E.M.; a, b: n≥5, and c, d: n=3 biological replicates) in worms was calculated, against control, unexposed, values (assigned a value of 1, dotted line) based on band intensity analysis after normalisation against actin. *p≤0.05, **p≤0.01, and ***p≤0.001 (ANOVA), compared to control values.

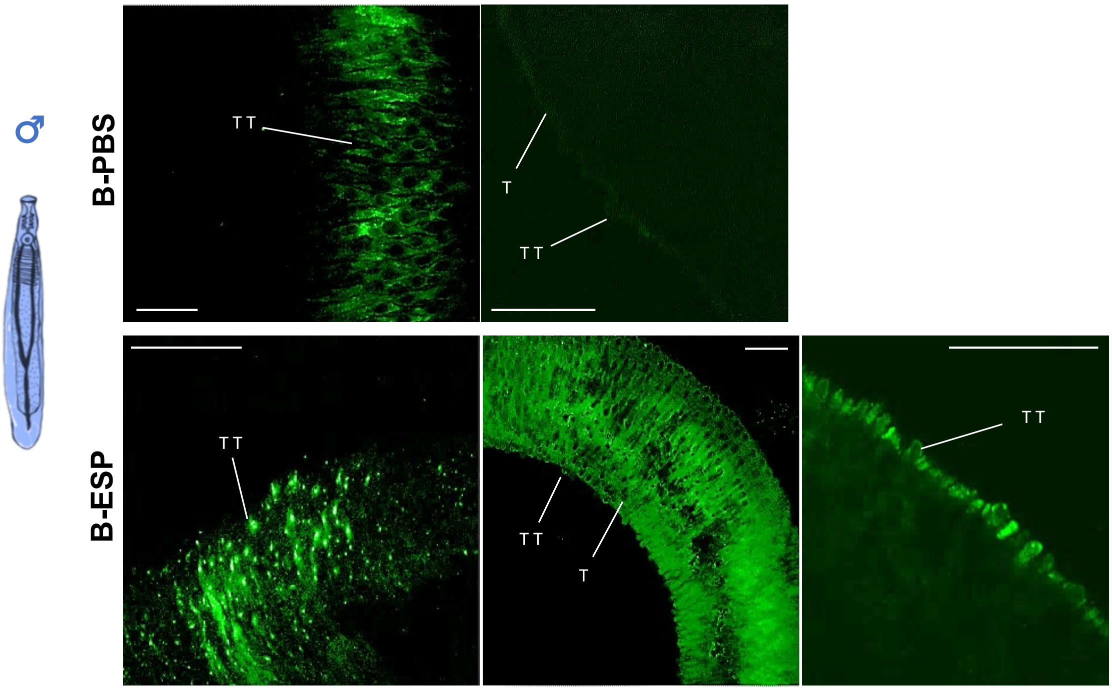

Supplementary Fig. 5 Adult female *S. mansoni* ESPs bind to the tegument tubercles and canyons between the tubercles of adult male worms. Male worms were exposed to female worm biotinylated ESPs (B-ESP), or PBS (B-PBS; control) for 15 min. Worms were then fixed, incubated in anti-biotin FITC (green) antibodies, and analysed by CLSM. Images shown are maximum projections of z series, except those showing sections through the tubercles, which are single z-scans. Images are representative of those from five individual worms. T, tegument; TT tegument tubercles. Scale bar = 50 µm.

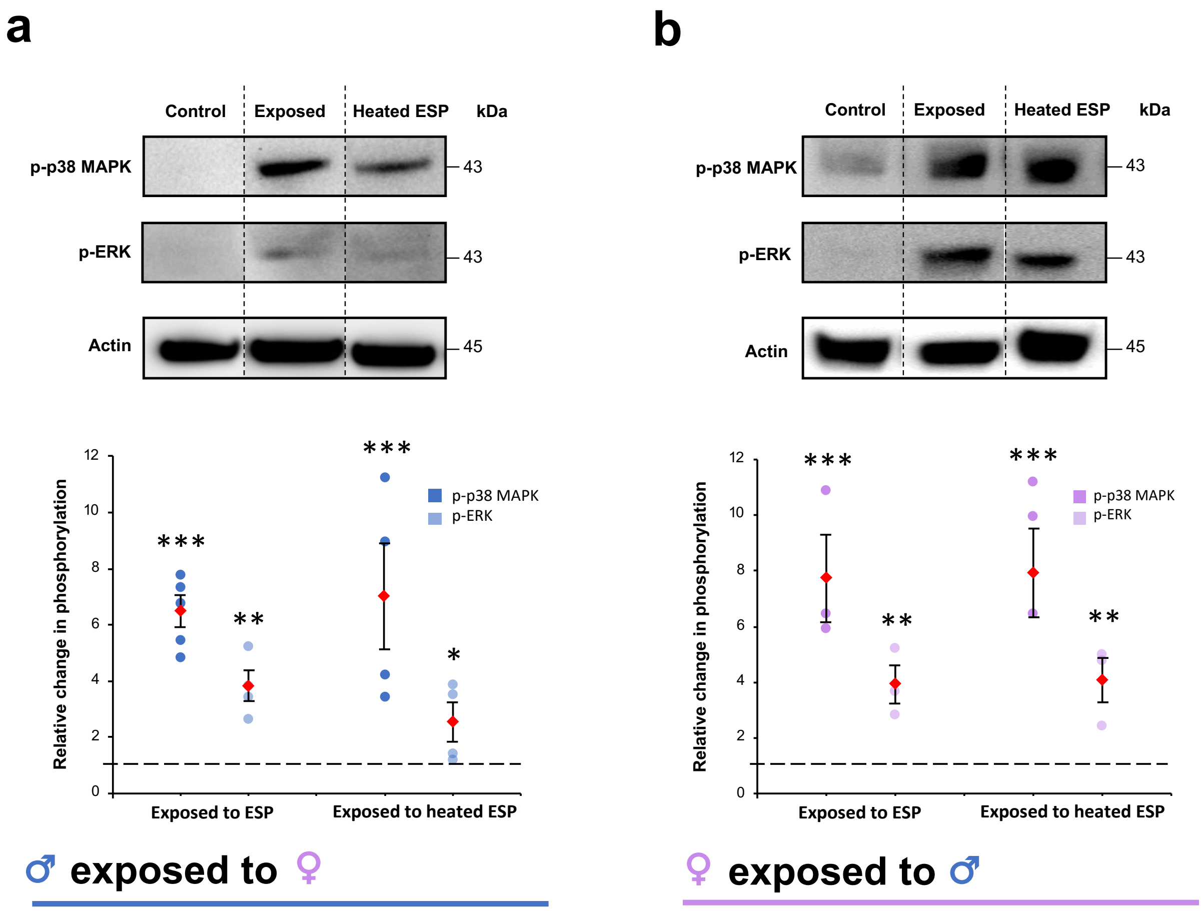

Supplementary Fig. 6 Heating adult *S. mansoni* ESPs does not prevent them from inducing p38 MAPK or ERK activation in opposite sex worms. a Male, or **b** female worms were exposed for 15 min to opposite sex worm culture media containing ESPs (exposed) or ESPs that had been heat treated (20 min at 95°C). Worm tegument proteins were extracted, and equal protein amounts processed for western blotting with anti-phospho-p38 MAPK or -ERK antibodies. Blots were also re-probed for actin as loading control. Vertical dotted lines on the blot images indicate non-adjacent lanes. Mean relative change in phosphorylation (±S.E.M.; n≥3 biological replicates) in worms was calculated against control 15 min unexposed values (assigned a value of 1, dotted line), based on band intensity analysis after normalisation against actin. *p≤0.05, **p≤0.01, and ***p≤0.001 (ANOVA), compared to control values.

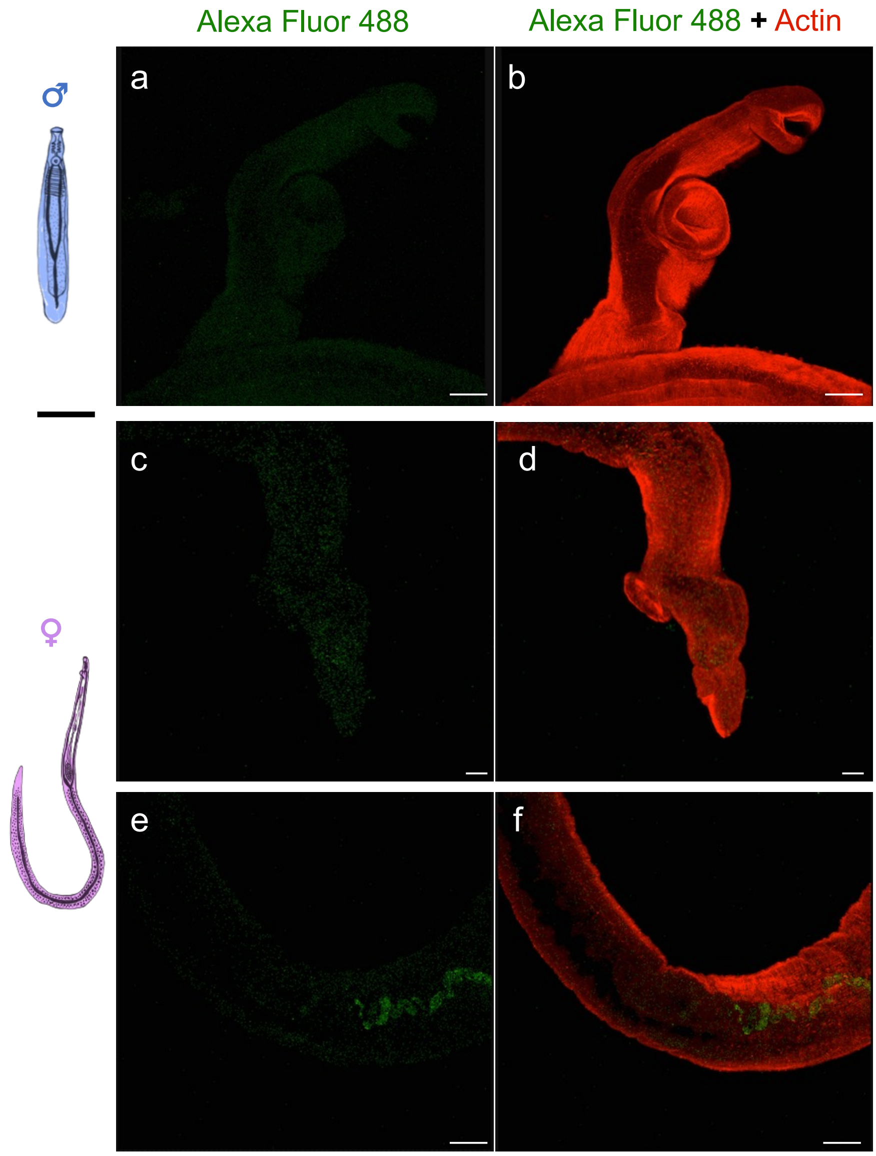

Supplementary Fig. 7 Negative control *S. mansoni* worms display minimal background staining for immunofluorescence. Adult male and female worms were incubated with Alexa Fluor 488 (green) secondary antibodies, and rhodamine phalloidin (red) to stain F-actin. **a-b** Male and **c-d** female worm anterior regions showing low Alexa Fluor 488 signal. **e-f** Female worm displaying region surrounding worm ovary and vitellaria with some low Alexa Fluor 488 signal in the vitellaria. All images are maximum projections of z-series and are representative of those from three individual worms of each sex. Scale bar = 50 µm.

a

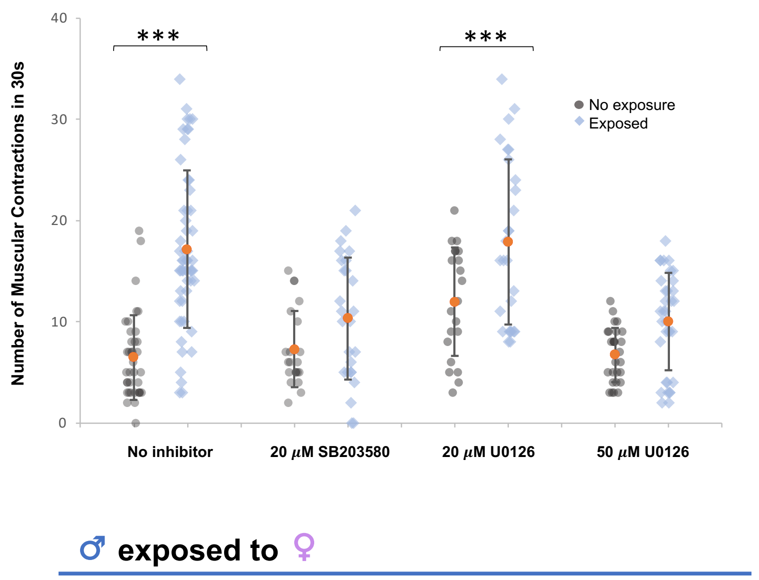

b

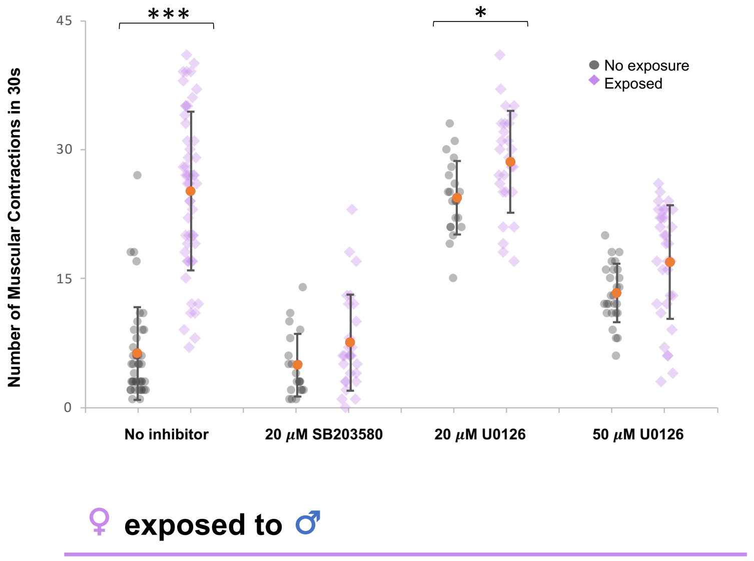

Supplementary Fig. 8 p38 MAPK and ERK inhibitors suppress *S. mansoni* adult worm hyperkinesia induced by opposite sex ESPs. a Adult male worms, or **b** adult female worms, cultured for 24 h, were either treated to SB203580 (20 µM) or U0126 (20 µM or 50 µM) for 1 h prior to being exposed to culture media containing ESPs from opposite sex adult worms; 30 s movies were captured after 3 min to evaluate the number of gross muscular contractions made within 30 s for each treatment. Individual and mean values are shown (±S.D.; n=15 worms per treatment from at least two independent experiments); *p≤0.05, ***p≤0.001 (ANOVA), compared to worms not exposed to ESPs. Representative movies are shown in Supplementary Movie 1.

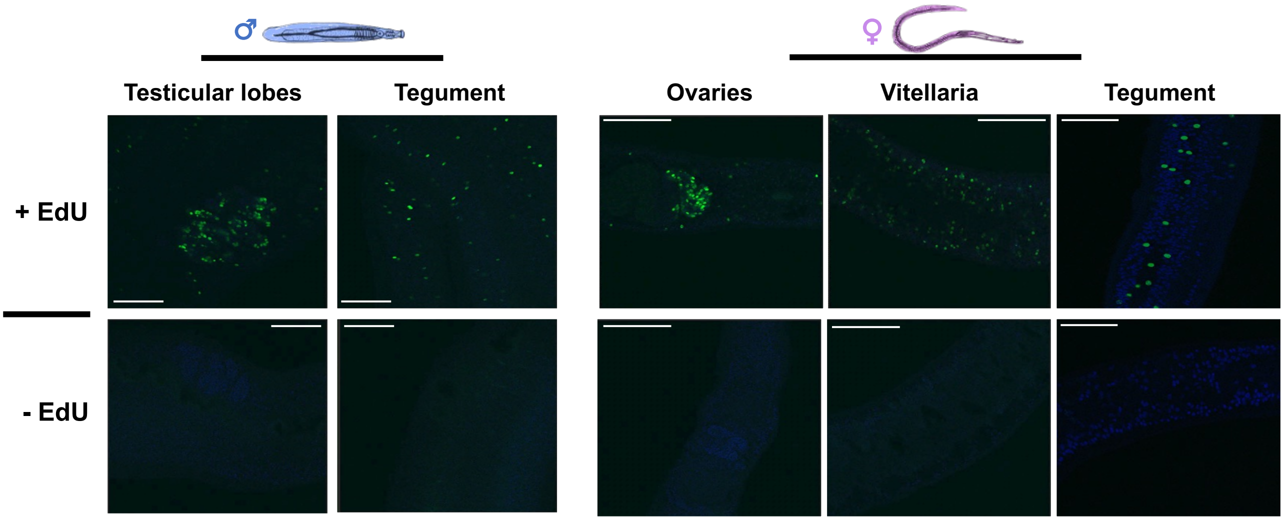

Supplementary Fig. 9 Alexa Fluor 488 azide dye does not stain adult *S. mansoni* stem cells in the absence of the EdU chase. Adult male and female worms were processed for EdU assay with and without the 24 h EdU chase. Worms were stained with DAPI and images captured by CLSM. Images shown maximum projections (50, 25, and 20 z-sections for the testes and ovaries, vitellaria, and teguments, respectively) and are representative of those observed in worms across at least three independent experiments. Bar = 50 µm; green = EdU^+ proliferating cells.

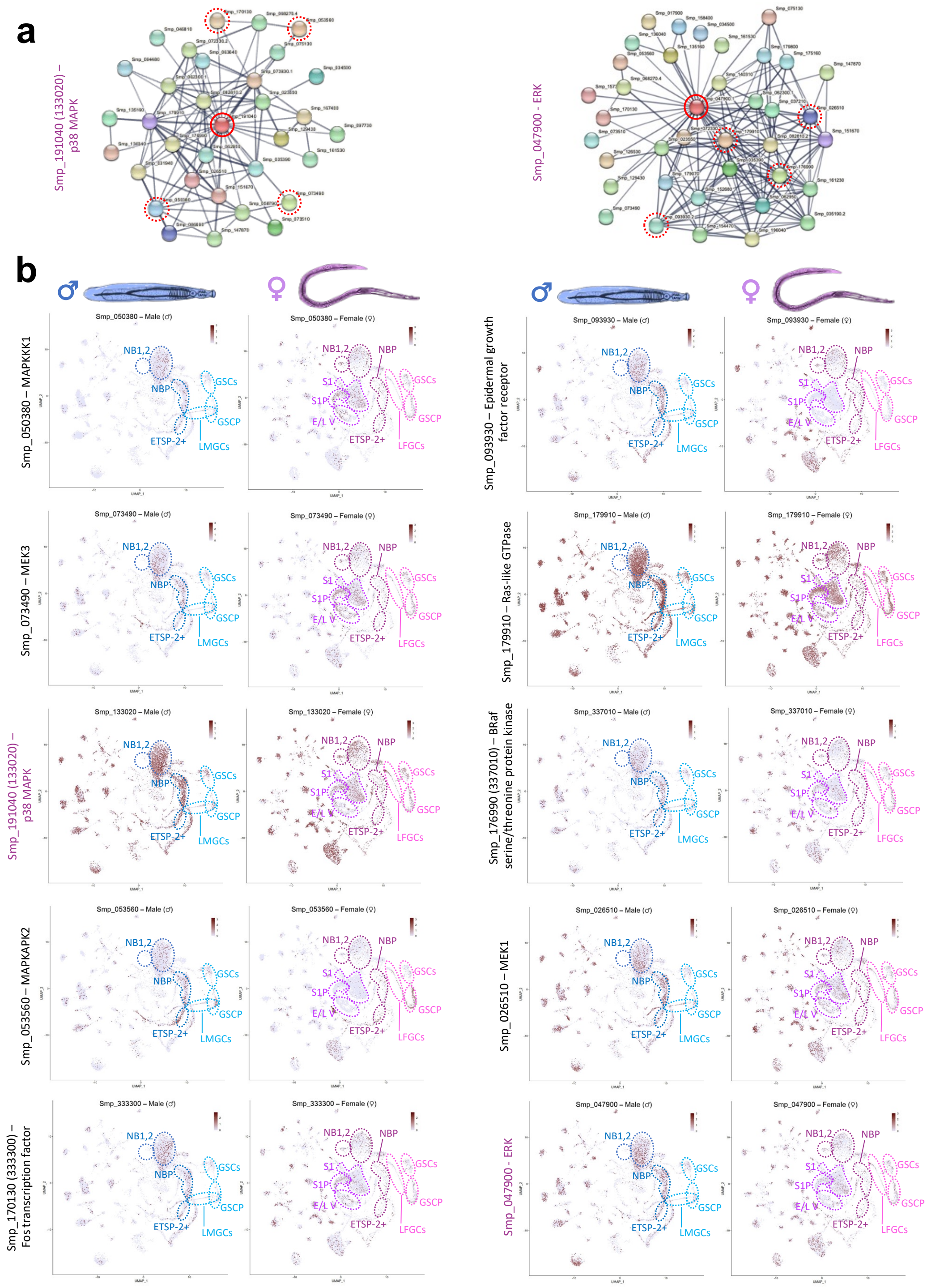

Supplementary Fig. 10 Multiple p38 MAPK and ERK pathway components are expressed in the neoblast and germinal stem cell populations of *S. mansoni*, including those that give rise to the tegument. a Putative physical/functional protein-protein interaction networks for p38 MAPK (Smp_ 191040) and ERK (Smp_047900) generated using STRING database. First shell high confidence (STRING global score >0.70) associations are shown (thicker inter-node lines = higher confidence), query proteins are in the centre of the networks (circled red). **b** Gene expression of p38 MAPK, ERK, and high confidence selected upstream regulatory and downstream target pathway partners (dotted circles in ‘a’) in adult mature worms. Uniform manifold approximation and projection (UMAP) plots highlighting cell clusters derived and adapted from single-cell RNA-seq data available at SchistoCyte Atlas (https://www.collinslab.org/schistocyte/) and queried for the network ‘Smp’ identifiers shown; new identifiers provided where previous (STRING) identifiers are mapped to new ‘Smp’ numbers at WormBase ParaSite (www.wormbase.org). NB1,2, neoblasts 1, 2; NBP, neoblast progeny; ETSP-2+, early TSP-2+ (tegument progenitor cells); GSCs, germinal stem cells; GSCP, germinal stem cell progeny; LFGCs, late female germinal cells; LMGCs, late male germ cells; S1, S1 cells; S1P, S1 progeny; E/L V, early/late vitellocytes.

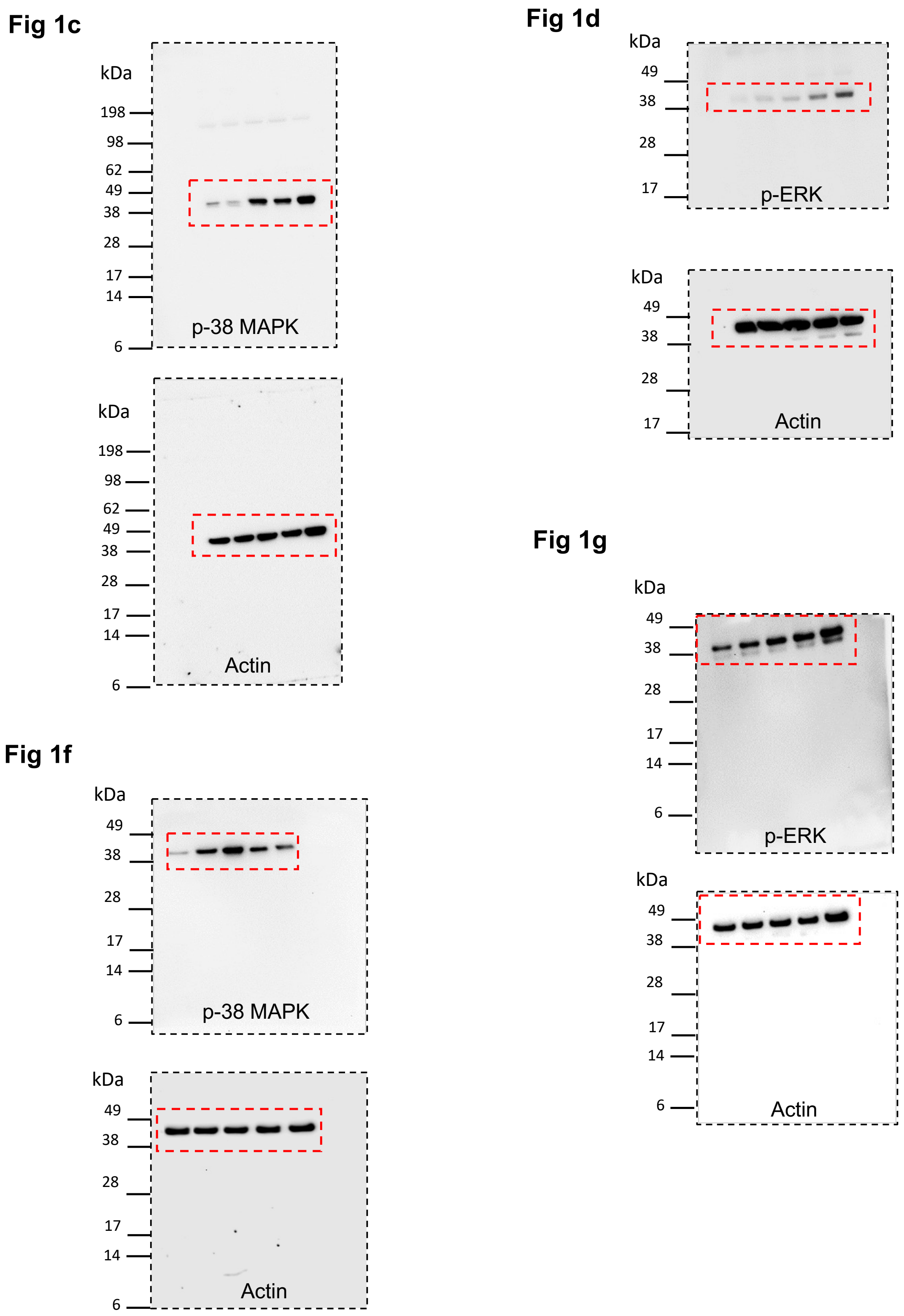

Supplementary Fig. 11 Western blots for Fig. 1. The red box highlights the region of immunoreactivity on each blot used in the main figure panels as indicated. Certain blots (Fig. 1d, f, g) were cut horizontally after Ponceau S staining to enable incubation in different primary antibodies, blots were also cut vertically to conserve antibody. The relative positions of the molecular weight markers (kDa) are shown.

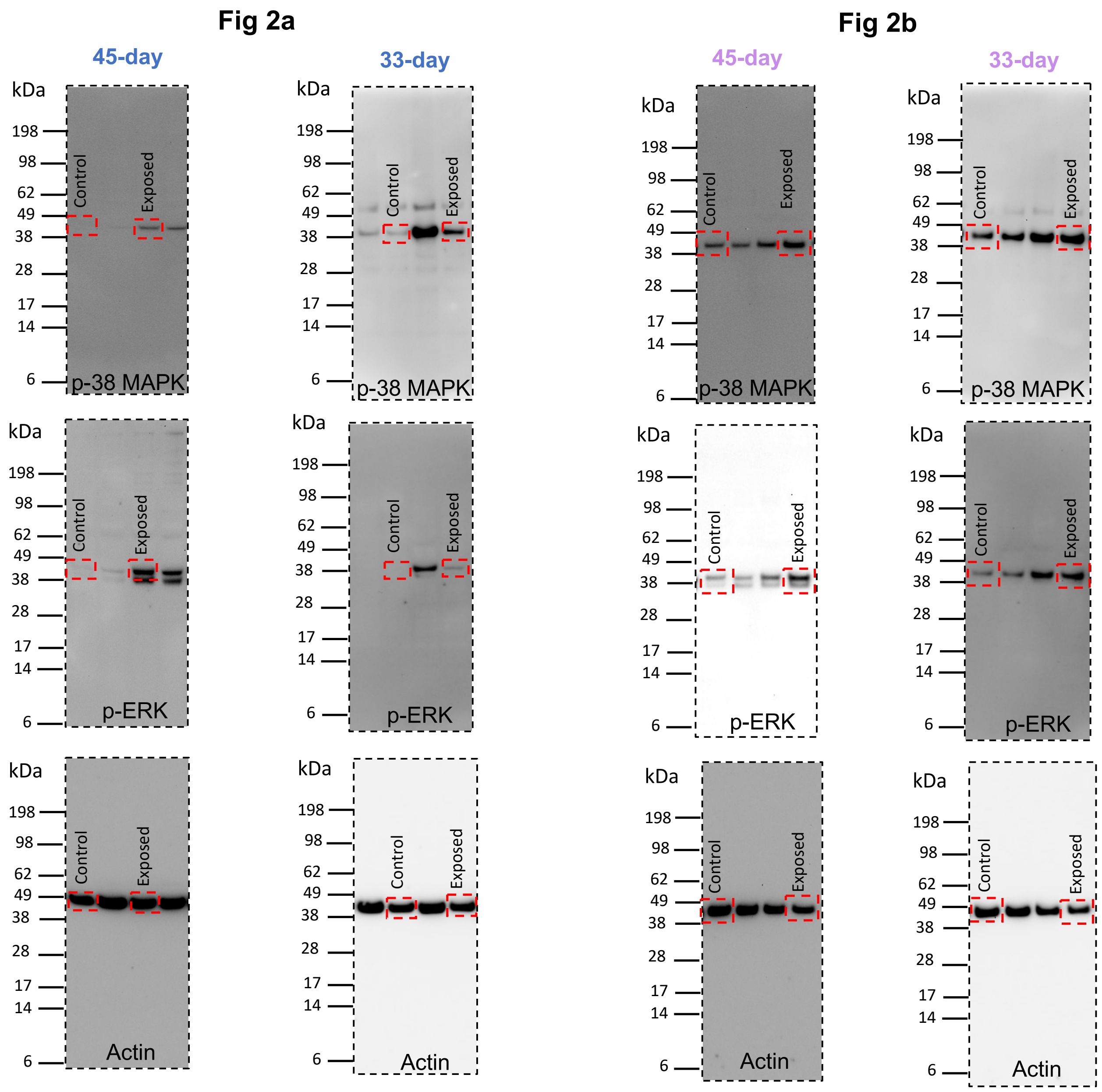

Supplementary Fig. 12 Western blots for Fig. 2. The red boxes highlight the regions of immunoreactivity on each blot used in the main figure panels as indicated. Blots were cut vertically after Ponceau S staining to enable incubation in different primary antibodies or to conserve antibody. The relative positions of the molecular weight markers (kDa) are shown.

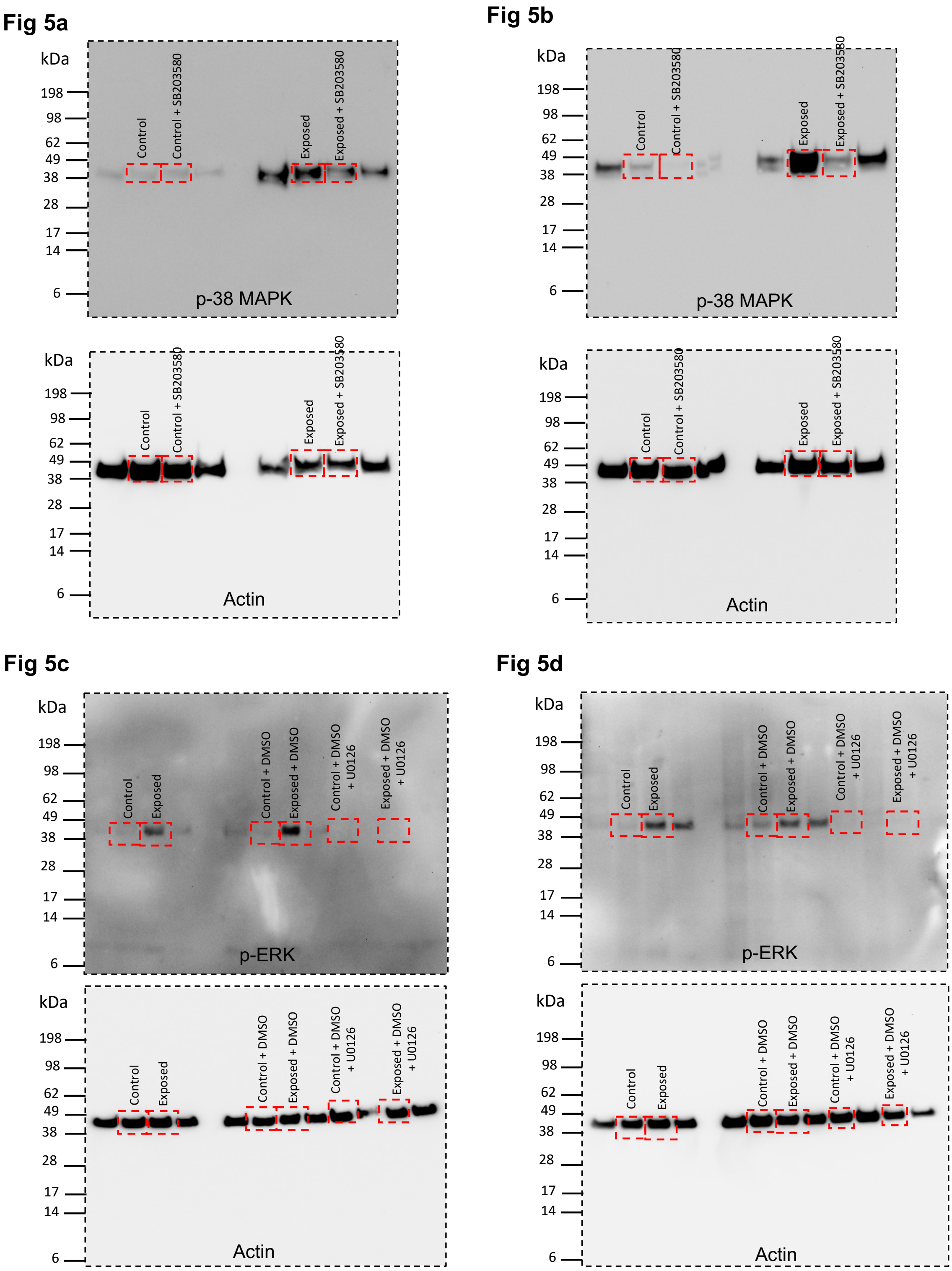

Supplementary Fig. 13 Western blots for Fig. 5. The red boxes highlight the regions of immunoreactivity on each blot used in the main figure panels as indicated. The relative positions of the molecular weight markers (kDa) are shown.

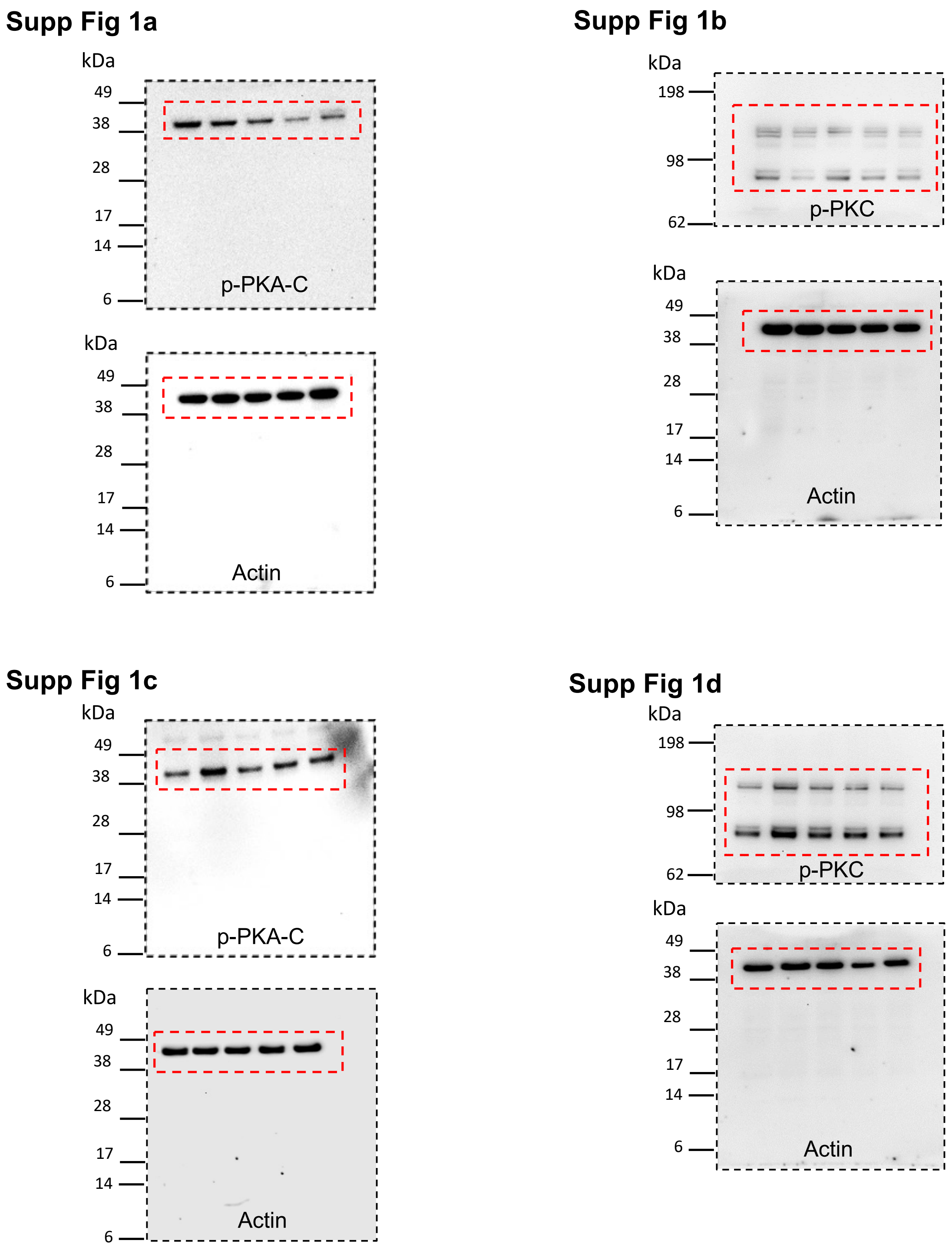

Supplementary Fig. 14 Western blots for Supplementary Fig. 1 (a-d). The red box highlights the region of immunoreactivity on each blot used in the main figure panels as indicated. All blots were cut horizontally after Ponceau S staining to enable incubation in different primary antibodies; some were cut vertically to conserve antibody. The relative positions of the molecular weight markers (kDa) are shown.

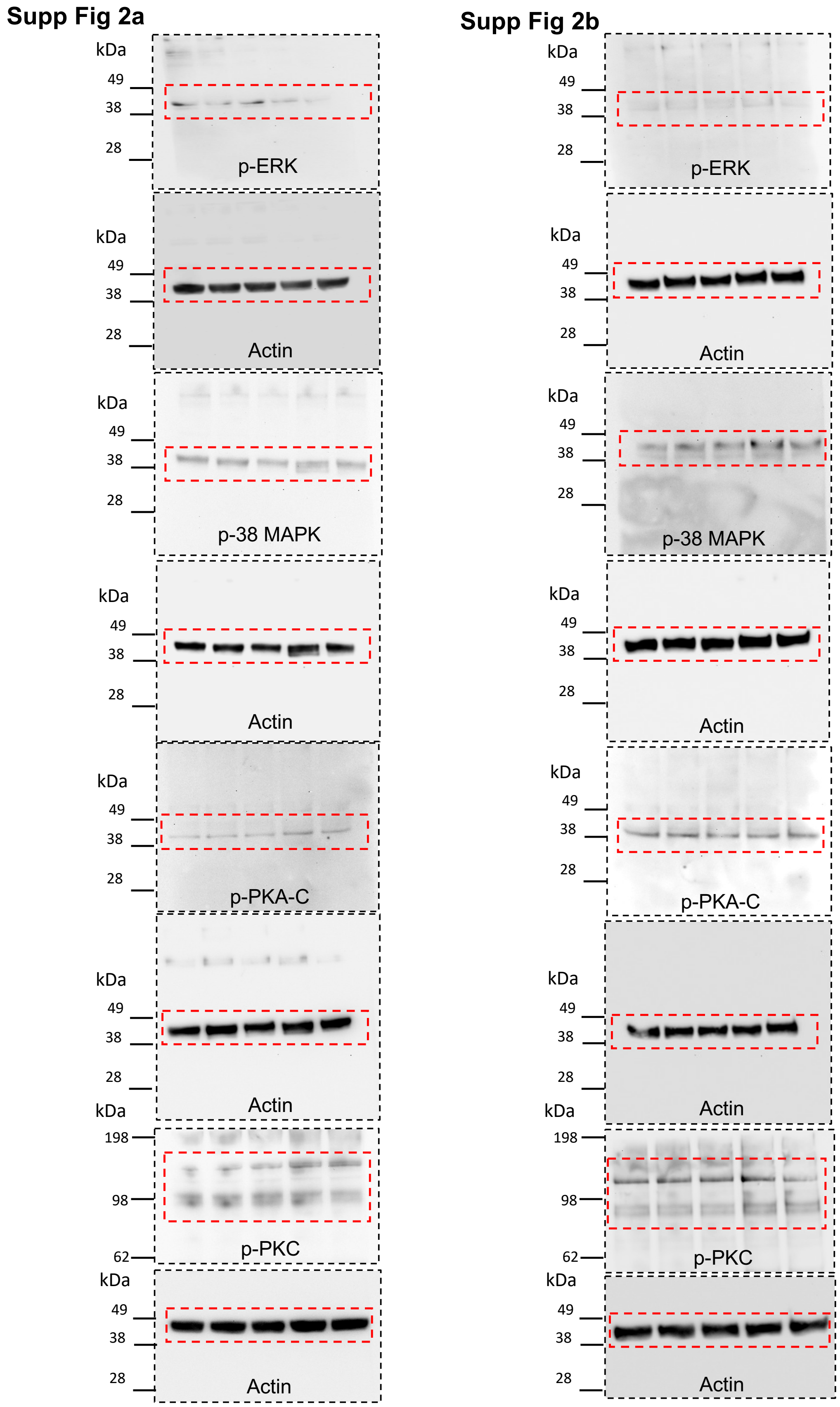

Supplementary Fig. 15 Western blots for Supplementary Fig. 2 (a, b). The red box highlights the region of immunoreactivity on each blot used in the main figure panels as indicated. All blots were cut horizontally and vertically after Ponceau S staining to enable incubation in different primary antibodies and to conserve antibody. The relative positions of the molecular weight markers (kDa) are shown.

Supp Fig 3a

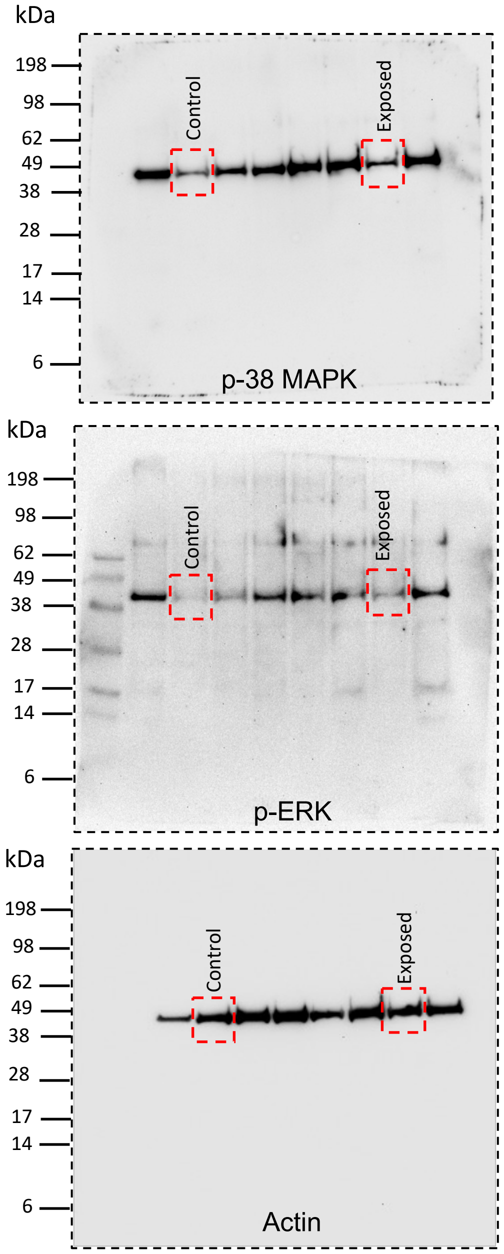

Supp Fig 3b

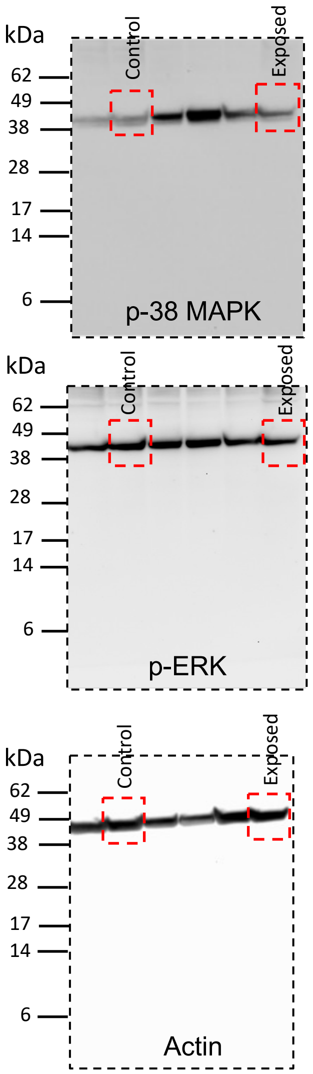

Supplementary Fig. 16 Western blots for Supplementary Fig. 3 (a, b). The red boxes highlight the regions of immunoreactivity on each blot used in the main figure panels as indicated. Certain blots (Supp Fig 3b) were cut horizontally and vertically after Ponceau S staining. The relative positions of the molecular weight markers (kDa) are shown.

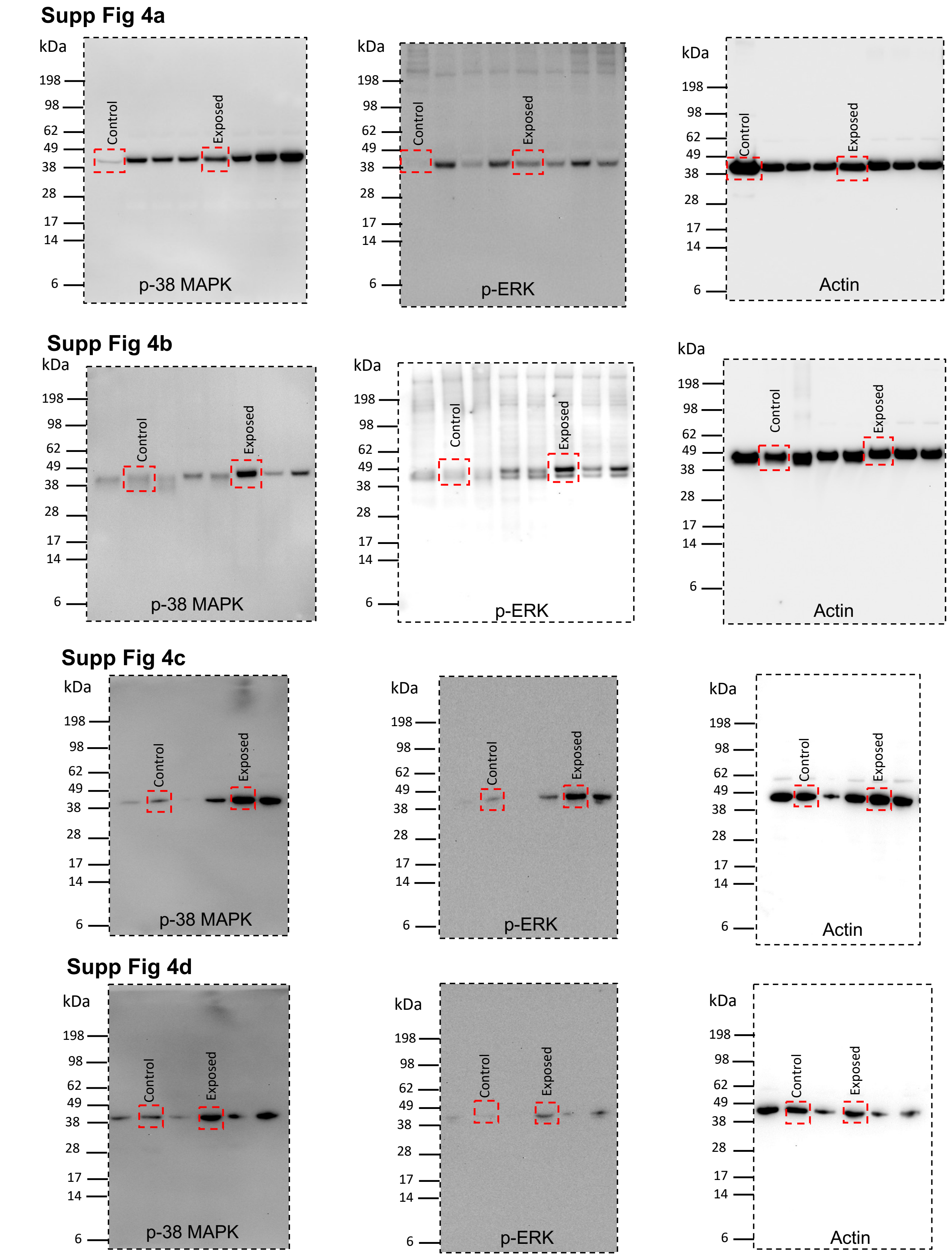

Supplementary Fig. 17 Western blots for Supplementary Fig. 4 (a-d). The red boxes highlight the regions of immunoreactivity on each blot used in the main figure panels as indicated. Blots were cut vertically after Ponceau S staining to enable incubation in different primary antibodies or to conserve antibody. The relative positions of the molecular weight markers (kDa) are shown.

Supp Fig 6a

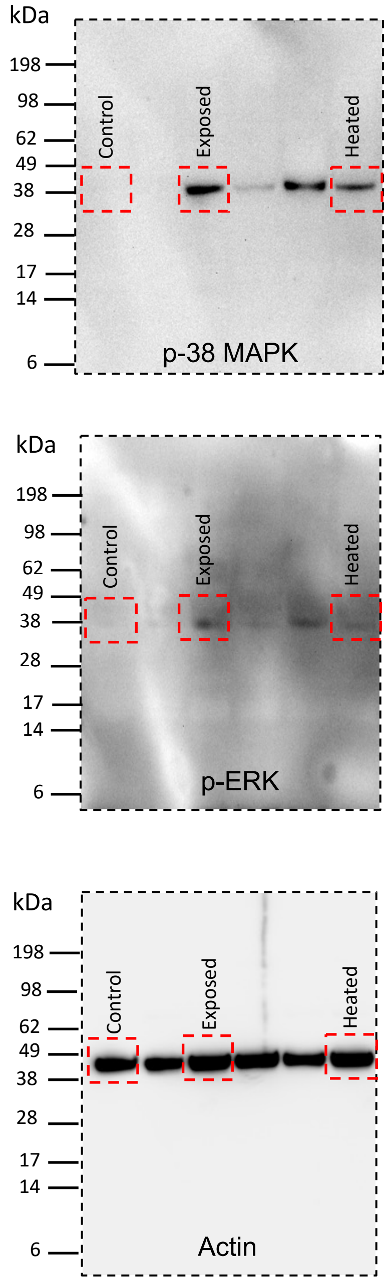

Supp Fig 6b

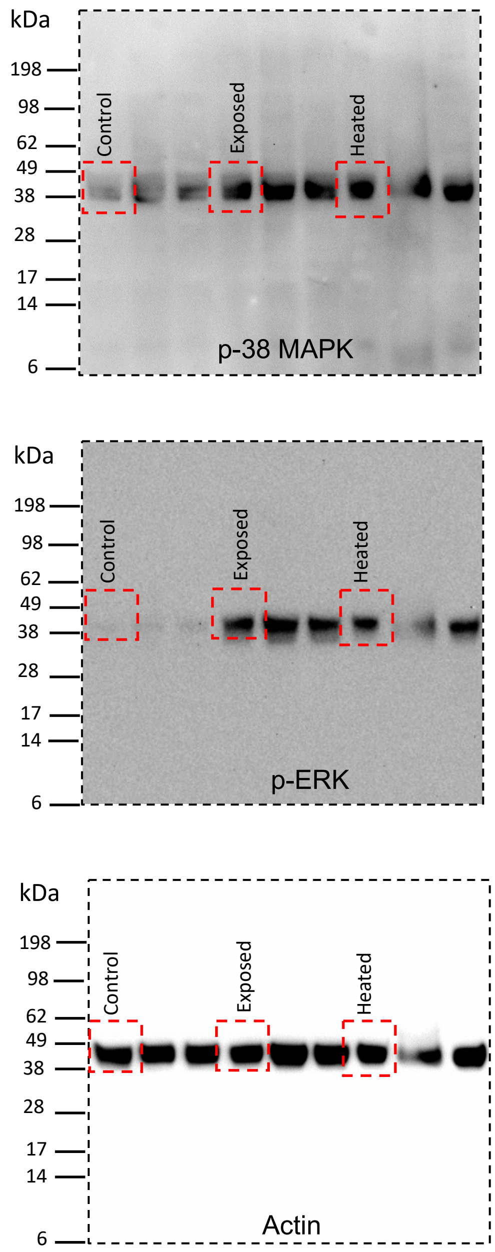

Supplementary Fig. 18 Western blots for Supplementary Fig. 6 (a, b). The red boxes highlight the regions of immunoreactivity on each blot used in the main figure panels as indicated. Certain blots (Supp Fig 6a) were cut vertically after Ponceau S staining. The relative positions of the molecular weight markers (kDa) are shown.
