# Supplementary material for: Schistosoma mansoni excretory-secretory products induce protein kinase signalling, hyperkinesia, and stem cell proliferation in the opposite sex
Source: Commun Biol. 2023 Sep 26;6:985. doi: 10.1038/s42003-023-05333-9 (PMC10522684; doi:10.1038/s42003-023-05333-9)
Supplement: Supplementary file 2 — Description of Additional Supplementary Files [file 42003_2023_5333_MOESM2_ESM.pdf]

## Description of Additional Supplementary Files

**File name:** Supplementary Movie 1
**Description:** Representative movies showing the effect of the various treatments on adult worm motility at 3 min after ESP exposure. Adult male worms, or adult female worms, cultured for 24 h were exposed to media containing ESPs from opposite sex adult worms or left untreated, and 30 s-movies recorded at indicated time points. Worms were also incubated in 20 μM SB203580, 20 μM U0126, or 50 μM U0126 for 1 h prior to ESP exposure.

**File name:** Supplementary Data 1
**Description:** Source data for graphs in the paper.
