# Supplementary material for: Schistosoma mansoni excretory-secretory products induce protein kinase signalling, hyperkinesia, and stem cell proliferation in the opposite sex
Source: Commun Biol. 2023 Sep 26;6:985. doi: 10.1038/s42003-023-05333-9 (PMC10522684; doi:10.1038/s42003-023-05333-9)
Supplement: Supplementary file 5 — Reporting Summary [file 42003_2023_5333_MOESM5_ESM.pdf]

nature portfolio

Corresponding author(s): Anthony J Walker

Last updated by author(s): 21/8/23

Reporting Summary

Nature Portfolio wishes to improve the reproducibility of the work that we publish. This form provides structure for consistency and transparency in reporting. For further information on Nature Portfolio policies, see our Editorial Policies and the Editorial Policy Checklist.

Statistics

For all statistical analyses, confirm that the following items are present in the figure legend, table legend, main text, or Methods section.

n/a	Confirmed	
☐	☒	The exact sample size (*n*) for each experimental group/condition, given as a discrete number and unit of measurement
☐	☒	A statement on whether measurements were taken from distinct samples or whether the same sample was measured repeatedly
☐	☒	The statistical test(s) used AND whether they are one- or two-sided<br>*Only common tests should be described solely by name; describe more complex techniques in the Methods section.*
☒	☐	A description of all covariates tested
☒	☐	A description of any assumptions or corrections, such as tests of normality and adjustment for multiple comparisons
☐	☒	A full description of the statistical parameters including central tendency (e.g. means) or other basic estimates (e.g. regression coefficient) AND variation (e.g. standard deviation) or associated estimates of uncertainty (e.g. confidence intervals)
☐	☒	For null hypothesis testing, the test statistic (e.g. *F*, *t*, *r*) with confidence intervals, effect sizes, degrees of freedom and *P* value noted<br>*Give P values as exact values whenever suitable.*
☒	☐	For Bayesian analysis, information on the choice of priors and Markov chain Monte Carlo settings
☒	☐	For hierarchical and complex designs, identification of the appropriate level for tests and full reporting of outcomes
☒	☐	Estimates of effect sizes (e.g. Cohen's *d*, Pearson's *r*), indicating how they were calculated

Our web collection on statistics for biologists contains articles on many of the points above.

Software and code

Policy information about availability of computer code

Data collection	No software was used
Data analysis	Minitab (Version 19) was used.

For manuscripts utilizing custom algorithms or software that are central to the research but not yet described in published literature, software must be made available to editors and reviewers. We strongly encourage code deposition in a community repository (e.g. GitHub). See the Nature Portfolio guidelines for submitting code & software for further information.

Data

Policy information about availability of data

All manuscripts must include a data availability statement. This statement should provide the following information, where applicable:

- Accession codes, unique identifiers, or web links for publicly available datasets
- A description of any restrictions on data availability
- For clinical datasets or third party data, please ensure that the statement adheres to our policy

All data that have been generated or analysed during this study are included in the published article and the associated supplementary files.

Human research participants

Policy information about studies involving human research participants and Sex and Gender in Research.

Reporting on sex and gender	Our manuscript does not involve human research participants
Population characteristics	*Describe the covariate-relevant population characteristics of the human research participants (e.g. age, genotypic information, past and current diagnosis and treatment categories). If you filled out the behavioural & social sciences study design questions and have nothing to add here, write "See above."*
Recruitment	*Describe how participants were recruited. Outline any potential self-selection bias or other biases that may be present and how these are likely to impact results.*
Ethics oversight	*Identify the organization(s) that approved the study protocol.*

Note that full information on the approval of the study protocol must also be provided in the manuscript.

Field-specific reporting

Please select the one below that is the best fit for your research. If you are not sure, read the appropriate sections before making your selection.

☒ Life sciences ☐ Behavioural & social sciences ☐ Ecological, evolutionary & environmental sciences

For a reference copy of the document with all sections, see nature.com/documents/nr-reporting-summary-flat.pdf

Life sciences study design

All studies must disclose on these points even when the disclosure is negative.

Sample size	No sample size calculation was performed. We know from prior experience of western blotting and functional assays with schistosomes how many replicates are usually required to demonstrate an effect, if that effect is of significant magnitude to be biologically relevant.
Data exclusions	No data obtained/analysed were excluded from the analyses. However, if a lane on a western blot displayed problems upon total Ponceau S staining then the sample was excluded.
Replication	All attempts at replication were successful. However, the magnitude of effect can vary between individual biological replicates and that is why we performed multiple replicates for each experiment as detailed in the manuscript legends etc.
Randomization	Parasites were allocated to experimental groups randomly.
Blinding	Blinding was not relevant to this study. It is not normal for investigators in cell biology research of this type to be blinded during data acquisition. It would also be difficult to do this routinely, as it is not always possible to be with my researcher in the laboratory to facilitate blinding of data.

Reporting for specific materials, systems and methods

We require information from authors about some types of materials, experimental systems and methods used in many studies. Here, indicate whether each material, system or method listed is relevant to your study. If you are not sure if a list item applies to your research, read the appropriate section before selecting a response.

Materials & experimental systems

n/a	Involved in the study	
☐	☒	Antibodies
☒	☐	Eukaryotic cell lines
☒	☐	Palaeontology and archaeology
☐	☒	Animals and other organisms
☒	☐	Clinical data
☒	☐	Dual use research of concern

Methods

n/a	Involved in the study	
☒	☐	ChIP-seq
☒	☐	Flow cytometry
☒	☐	MRI-based neuroimaging

Antibodies

Antibodies used	We used the following antibodies in this study: anti-phospho S/T/Y antibodies (Abcam, Ref - SPM101), anti-phospho -p38 (Thr180/

Antibodies used	Tyr182) (Cell Signalling Technology, Ref – 9215; 3D7), anti-phospho-ERK (p42/p44 MAPK) (Thr202/Tyr204) (Cell Signalling Technology, Ref – 9101), anti-phospho-PKA-C (Thr197) (Cell Signalling Technology, Ref – 4781), anti-phospho-PKC (pan) (Thr410) antibodies (Cell Signalling Technology, Ref – 2060; 190D10), horseradish peroxidase (HRP)-conjugated anti-rabbit secondary antibodies (Cell signalling Technology, Ref – 7074), HRP-conjugated anti-actin antibodies (Santa Cruz Biotechnology, Ref- sc-47778), Alexa Fluor 488 anti-rabbit secondary antibodies (Life Technologies, Ref - A-11034). We do not have the batch numbers for these antibodies unfortunately. The research was done over several years. We never saw differences in how the antibodies performed over time.
Validation	All of the citations and references to where the anti-phospho antibodies have been validated by us for use in Schistosoma mansoni have been written into the manuscript text. Importantly, these antibodies react exclusively with the phosphorylated (activated) form of each target kinase enabling the study of specific pathway activation following treatment. The references are as follows:<br>Hirst, N. L., Lawton, S. P. & Walker, A. J. Protein kinase A signalling in Schistosoma mansoni cercariae and schistosomules. Int J Parasitol 46, 425–437 (2016).<br>Ressurreição, M., Rollinson, D., Emery, A. M. & Walker, A. J. A role for p38 mitogen-activated protein kinase in early post-embryonic development of Schistosoma mansoni. Mol Biochem Parasitol 180, 51–55 (2011).<br>Ressurreição, M., Rollinson, D., Emery, A. M. & Walker, A. J. A role for p38 MAPK in the regulation of ciliary motion in a eukaryote. BMC Cell Biol 12, 6 (2011).<br>Ressurreição, M. et al. Sensory Protein Kinase Signaling in Schistosoma mansoni Cercariae: Host Location and Invasion. Journal of Infectious Diseases 212, 1787–1797 (2015).<br>de Saram, P. S. R. et al. Functional mapping of protein kinase A reveals its importance in adult Schistosoma mansoni motor activity. PLoS Negl Trop Dis 7, e1988 (2013).<br>Ressurreição, M. et al. Protein kinase C and extracellular signal-regulated kinase regulate movement, attachment, pairing and egg release in Schistosoma mansoni. PLoS Negl Trop Dis 8, e2924 (2014).<br>Ludtmann, M. H. R., Rollinson, D., Emery, A. M. & Walker, A. J. Protein kinase C signalling during miracidium to mother sporocyst development in the helminth parasite, Schistosoma mansoni. Int J Parasitol 39, 1223–1233 (2009).<br>Ressurreição, M. et al. Molecular characterization of host-parasite cell signalling in Schistosoma mansoni during early development. Sci Rep 6, 35614 (2016).<br>The remaining antibodies are generic and are secondary antibodies.

Animals and other research organisms

Policy information about studies involving animals; ARRIVE guidelines recommended for reporting animal research, and Sex and Gender in Research

Laboratory animals	Female Balb/c mice (Mus musculus), infected with Schistosoma parasites at 6 weeks old.
Wild animals	This study did not involve wild animals
Reporting on sex	Female Balb/c mice were only used to host the parasite, and these are routinely used for this in the laboratory. Studies were not done on the mice themselves only on the parasites derived from them.
Field-collected samples	The study did not involve samples collected from the field.
Ethics oversight	The Wellcome Sanger Institute and University of Nottingham

Note that full information on the approval of the study protocol must also be provided in the manuscript.
